# Muscle-Derived IL-6 Is Not Regulated by IL-1 during Exercise. A Double Blind, Placebo-Controlled, Randomized Crossover Study

**DOI:** 10.1371/journal.pone.0139662

**Published:** 2015-10-08

**Authors:** Thierry M. Nordmann, Eleonora Seelig, Katharina Timper, Mareike Cordes, Michael Coslovsky, Henner Hanssen, Arno Schmidt-Trucksäss, Marc Y. Donath

**Affiliations:** 1 Clinic of Endocrinology, Diabetes and Metabolism University Hospital Basel, and Department Biomedicine. University of Basel, 4031 Basel, Switzerland; 2 Division of Sports and Exercise Medicine, Department of Sport, Exercise and Health, Medical Faculty, University of Basel, Basel, Switzerland; 3 Clinical Trial Unit, University Hospital Basel, 4031 Basel, Switzerland; University of Milan, ITALY

## Abstract

Exercise increases muscle derived Interleukin–6 (IL–6) leading to insulin secretion via glucagon-like peptide–1. IL–1 antagonism improves glycemia and decreases systemic inflammation including IL–6 in patients with type 2 diabetes. However, it is not known whether physiological, exercise-induced muscle-derived IL–6 is also regulated by the IL–1 system. Therefore we conducted a double blind, crossover study in 17 healthy male subjects randomized to receive either the IL–1 receptor antagonist IL-1Ra (anakinra) or placebo prior to an acute treadmill exercise. Muscle activity led to a 2–3 fold increase in serum IL–6 concentrations but anakinra had no effect on this exercise-induced IL–6. Furthermore, the IL–1 responsive inflammatory markers CRP, cortisol and MCP–1 remained largely unaffected by exercise and anakinra. We conclude that the beneficial effect of muscle-induced IL–6 is not meaningfully affected by IL–1 antagonism.

**Trial Registration:**

ClinicalTrials.gov NCT01771445

## Introduction

Exercise improves glycaemia in patients with type 2 diabetes. This is due to multiple factors including increased calorie consumption and insulin independent glucose uptake in muscle. Furthermore, the active muscle produces several molecules that may have endocrine functions and contribute to the beneficial effect of exercise on metabolism[1, 2]. Indeed, in response to muscle contraction, IL–6 is released into the circulation in abundance [2]. Under physiological conditions, IL–6 appears to increase insulin sensitivity [3]. Furthermore, we have recently shown that IL–6 enhances glucagon-like peptide-1-mediated insulin secretion [4]. However, possibly due to the prevailing inflammation, in obese individuals IL–6 may have negative effects and precipitate insulin resistance [5].

Numerous observations and clinical studies have shown that inflammation has a substantial role in the pathogenesis of type 2 diabetes. In particular, pathological activation of IL–1 contributes to impaired insulin secretion and action [6]. Accordingly, IL–1 antagonism improves glycaemia and β-cell secretory function in patients with type 2 diabetes [7, 8]. Furthermore, IL–1 blockade reduces systemic inflammation including IL–6 [7, 8].

Little is known about the regulation of muscle-derived IL–6 during exercise. Although it seems to be independent of the nuclear factor 'kappa-light-chain-enhancer' of activated B-cells (NFκB)-pathway [9], it is not known whether IL–1 regulates muscle derived IL–6 during contraction. Therefore, the aim of this study was to investigate whether exercise-induced IL–6 is dependent on the IL–1 system. Furthermore, because IL–1β is linked to fatigue in patients with type 2 diabetes [10] and stimulates the hypothalamic-pituitary-adrenal axis [11], fatigue and cortisol levels were also studied.

## Materials and Methods

### Study design

The clinical study was designed as a double-blind, randomized, placebo-controlled, crossover, single-center study. Patient recruitment and all follow up visits were performed from November 2011 to May 2013 at the University Hospital Basel, Switzerland in accordance with the ICH-GCP guidelines and the Declaration of Helsinki, and approved by the Ethics Committee of Basel (Ref. 294/10) and Swissmedic (Ref. Nr. 2011DR1084). The study was registered on clinicaltrials.gov (NCT01771445). Because this study is a mechanistic study and not a treatment study, we realized only with a 3-month delay that it had to be registered. The authors confirm that all ongoing and related trials for this drug/intervention are registered. Written informed consent was obtained from all participants before study inclusion. The sample size was based on clinical and practical considerations.

### Study participants

Subjects were eligible for the study if they were male, apparently healthy, non-smoking, aged between 20 and 50 years with a body mass index between 18 and 26 kg/m^2^. Further inclusion criteria were regular exercise including a minimum of two runs weekly with a total duration of more than 2 hours.

Subjects were excluded if they showed clinical signs of infection, impaired fasting plasma glucose of more than 5.5 mmol/L, hematologic, renal, hepatic, cardiac, pulmonary or inflammatory disease, history of carcinoma or tuberculosis, increased alcohol consumption, known allergy to anakinra and current treatment with any drug. Subjects were not eligible for the study if they had used any investigational drug within 30 days prior to enrollment or within 5 half-lives of the investigational drug, whichever was longer.

### Treatment Assignment and Blinding

Once screening was completed and subject eligibility was confirmed, a subject was assigned a subject number randomly assigned to receive study medication. The Clinical Trial Unit of the University Hospital Basel, Switzerland, was responsible for treatment blinding and preparation of trial drugs throughout the study.

### Study procedure

The study consisted of one screening visit followed by 2 study visits separated by 7 days, and a follow up visit.

At the screening visit, a physical and laboratory examination, and an ECG were performed. Body composition was assessed using the Body Impedance Analyzer (Bodyimpedance Analyzer Model BIA 101, Akern Srl Florence Italy). A treadmill ergometer test was performed determining individual heart rate-oxygen consumption (VO_2_) relationships, and VO_2_max on which the exercise load for the acute exercise bout was based. Once eligible, patients were allocated according to a randomization list created by a biostatistician unrelated to the study. Patients as well as study personnel were blinded to the medication allocation.

For the 2 study visits, subjects were requested to fast 6–10 hours prior to the visit and were then asked to fill in an Activity Induced Fatigue Scale [12] followed by the Symbol Digit Modalities Test, a cognitive screening-test to evaluate information processing speed and working memory [13] and the Beck-Depression-Inventory Fast Screen to evaluate the impact of fatigue on cognitive, motoric, and emotional behavior [13]. Afterwards an intravenous catheter for blood drawings was placed in the forearm. 60 minutes before the start of the exercise bout and right after the first blood sample was taken, subjects received a single subcutaneous injection of 100mg anakinra (Kineret®) or placebo in a double-blind, crossover manner. At time 0, the subject started to run with a 5 minutes warm up period at 2 to 4 km/h at an incline of 0.5%. The treadmill speed was then increased to 75% of VO_2max_ based on heart rate measurements for 60 minutes followed by a “cool down” at walking speed for 5 minutes. 60 minutes after exercise the intravenous catheter was removed. In total, blood was drawn at 12 time points: 60 minutes before exercising (-60min.), every ten minutes starting immediately prior to the exercise until immediately after (0, 10, 20, 30, 40, 50, 60min.) and four times within the hour following the exercise (+10aE, 20aE, 30aE, 60aE). 1–2 hours after the end of the exercise bout, the study participants were asked again to fill in the fatigue, processing and emotional tests. The same procedures were performed one week after the first visit followed by a safety visit after an additional week.

### Study endpoints

Primary outcome measure was change in exercise-stimulated IL–6 plasma levels after administration of placebo and anakinra.

Secondary outcome measures were change in plasma levels of glucose, cortisol, inflammatory markers (high-sensitive (hs)-CRP, IL–8, monocyte chemotactic protein 1 (MCP–1)), and creatine kinase, as well as fatigue, information processing speed and working memory, and depression/ cognitive, motoric and emotional features.

### Sample collection and analytic procedure

Blood was collected into prechilled tubes that were immediately centrifuged at 4°C and aliquoted. All samples were immediately frozen and stored at -80°C until measurement. IL–6, IL–8, IL-1Ra, keratinocyte-derived chemokine (KC) and MCP–1 were measured using an electrochemiluminescence immunoassay according to the manufacturer’s instructions (Mesoscale Discovery [MSD], Gaithersburg, MD, USA). MSD plates were analyzed on a Sector™ MSD 2400 instrument and data were analyzed using DISCOVERY WORKBENCH 4.0 software. Measurements of hs-CRP, cortisol, and glucose were performed by automated biochemical analyses in the University Hospital Central Laboratories.

### MIN6B1 cell cultures

MIN6B1 cells were kindly provided by Dr. Philippe Halban (University of Geneva, Geneva, Switzerland) with permission from Dr. Junichi Miyazaki, University of Osaka. 3.5 × 10^4^ cells per well were seeded in 96-well plates and cultured in DMEM supplemented with 15% fetal bovine serum (FBS), 100 U/mL penicillin, 100 μg/mL streptomycin for 48 hours. Cells were subsequently incubated with 20v/v % of patient serum obtained at time point -60 and 40 min. after anakinra injection or after placebo from one study participant, in combination with 0.02 ng/ml recombinant mouse IL–1β (rmIL–1β; R&D Systems, Abingdon, UK) for 24 hours. Supernatant was harvested and KC levels were analyzed as outlined above.

### Statistical analysis

Descriptive measurements are reported as median and interquartile range.

For the pre-exercise time, the absolute change in biomarker measurement was calculated by detracting the values at injection time (-60 min.) from those at exercise begin (0 min.) in each period and modeling them.

To analyze the repeated measurements during exercise, linear mixed-effects models (LMM) were utilized. Endpoints were log-transformed in models where heteroscedasticity was detected. All models included study period as a fixed effect, to account for the crossover design. The interaction of study period and treatment was tested in all models; however, since it never was significant for biomarker models (all p >0.33), it was removed from all biomarker models. Treatment arm and time were included as fixed effects in the models. To test for linear and quadratic trends in time, time and time-squared were entered as continuous variables centered on mean exercise time (30 min.). The interactions between both time terms and the treatment-arm were first included in models, and when not significant, removed to make interpretation of main effects easier. To account for non-independence of measurements from the same subject, subject ID was included as a random effect in all models. In addition, models with >2 time points of measurements included an auto-regressive correlation structure of order 1 (AR1) to account for the correlation between observations measured close to each other. Least square means (predicted marginal values) and confidence intervals were calculated at specific time points for post-hoc tests. Marginal p-values are reported.

Fatigue and depression-indices measurements were analyzed similarly as described above, except that, since having only two measurements per endpoint, an ANCOVA approach was used. The post-exercise measurement was modeled with the pre-exercise measurement as a covariate and including study period, treatment and their interaction as fixed effects. As above, subject ID was included as a random effect in the models.

Analysis was performed using R—3.2.0 (R Core Team, 2015). Mixed effects models were fit using the R-package nlme (Pinheiro & Bates, 2000). LS means were calculated using the lsmeans package (Lenth & Hervé, 2015).

## Results

### Participant flow

**Fig 1:**
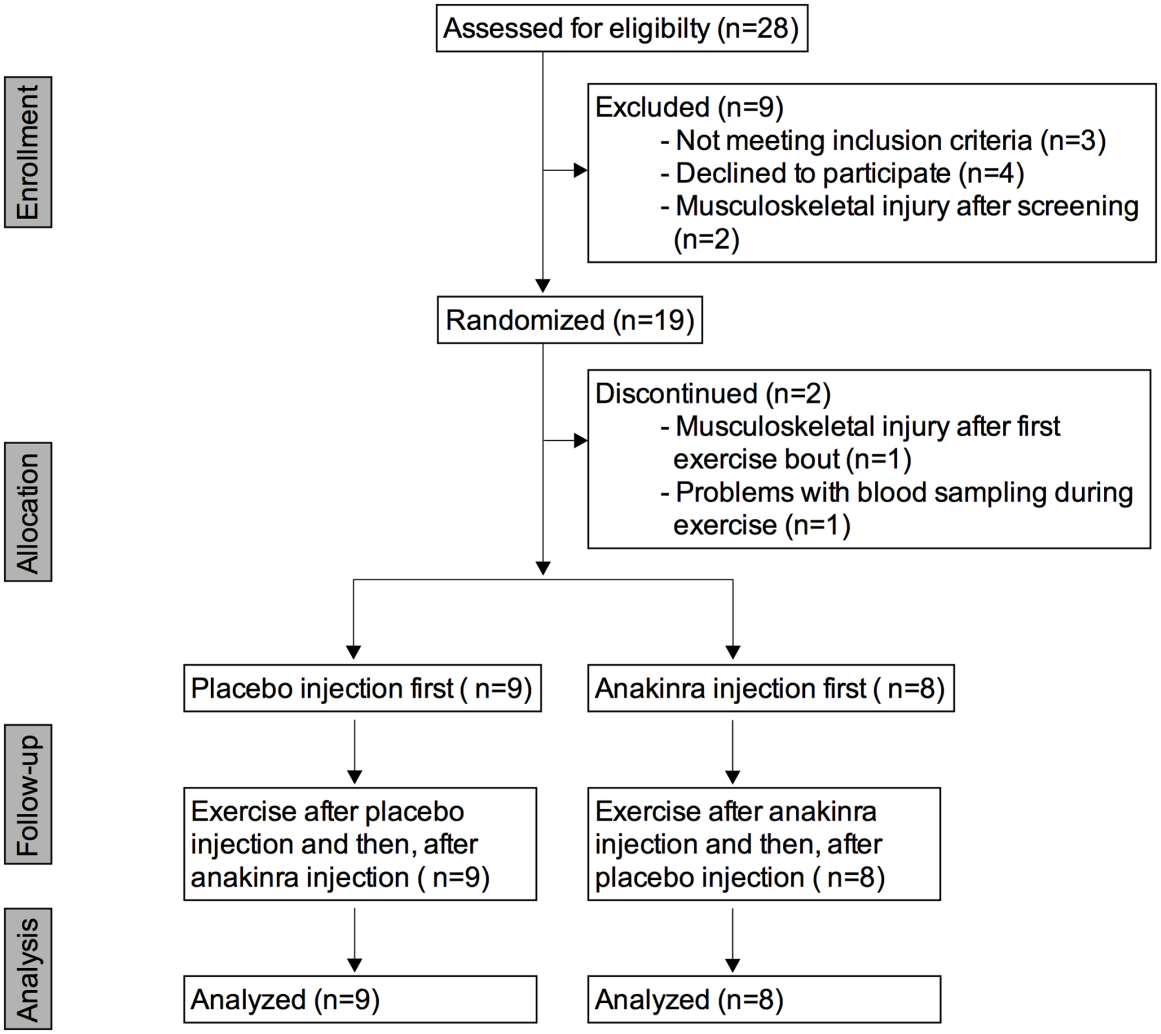
Study flowchart and CONSORT diagram.

Out of 19 subjects enrolled in the study 17 completed the study and were analyzed (Fig 1). Two subjects dropped out after the first exercise bout, one due to the difficulties due to blood sampling during the exercise and one due to a musculoskeletal injury after the visit.

### Baseline characteristics

Median age was 25 [23, 32] years and median body mass index was 23 [21, 25] kg/m^2^ (Table 1). The individuals studied reflected a typical population of apparently healthy Swiss people of the same age and body mass index.

**Table 1:** Baseline characteristics of study participants. Data represent the median and interquartile range, n = 17 subjects.

Characteristics	
Age (years)	25 [23, 32]
Pulse (b/min.)	63 [54, 73]
Blood pressure systolic (mmHg)	125 [116, 134]
Blood pressure diastolic (mmHg)	72 [68, 78]
Body mass index (kg/m^2^)	23 [21, 25]
Fat free mass (kg; n = 13)	39 [35, 40]
Body cell mass (kg; n = 13)	23 [20, 25]
Muscle mass (% weight; n = 13)	65 [59, 74]
Fat mass (kg/m; n = 13)	3 [1, 7]
Basal metabolic rate (kcal; n = 13)	1930 [1830, 2035]
Creatinine (μmol/l)	77 [73, 81]
Aspartat aminotransferase (U/l)	24 [21, 29]
Alanine aminotraμnsferase (U/l)	18 [16, 25]
High sensitive CRP (mg/l)	0.6 [0.3, 1]
Leucocytes (x10^9/l)	6 [5, 6]
Haemoglobin (g/l)	151 [143, 149]
Thrombocytes (x10^9/l)	249 [232, 296]

### IL–6

**Fig 2:**
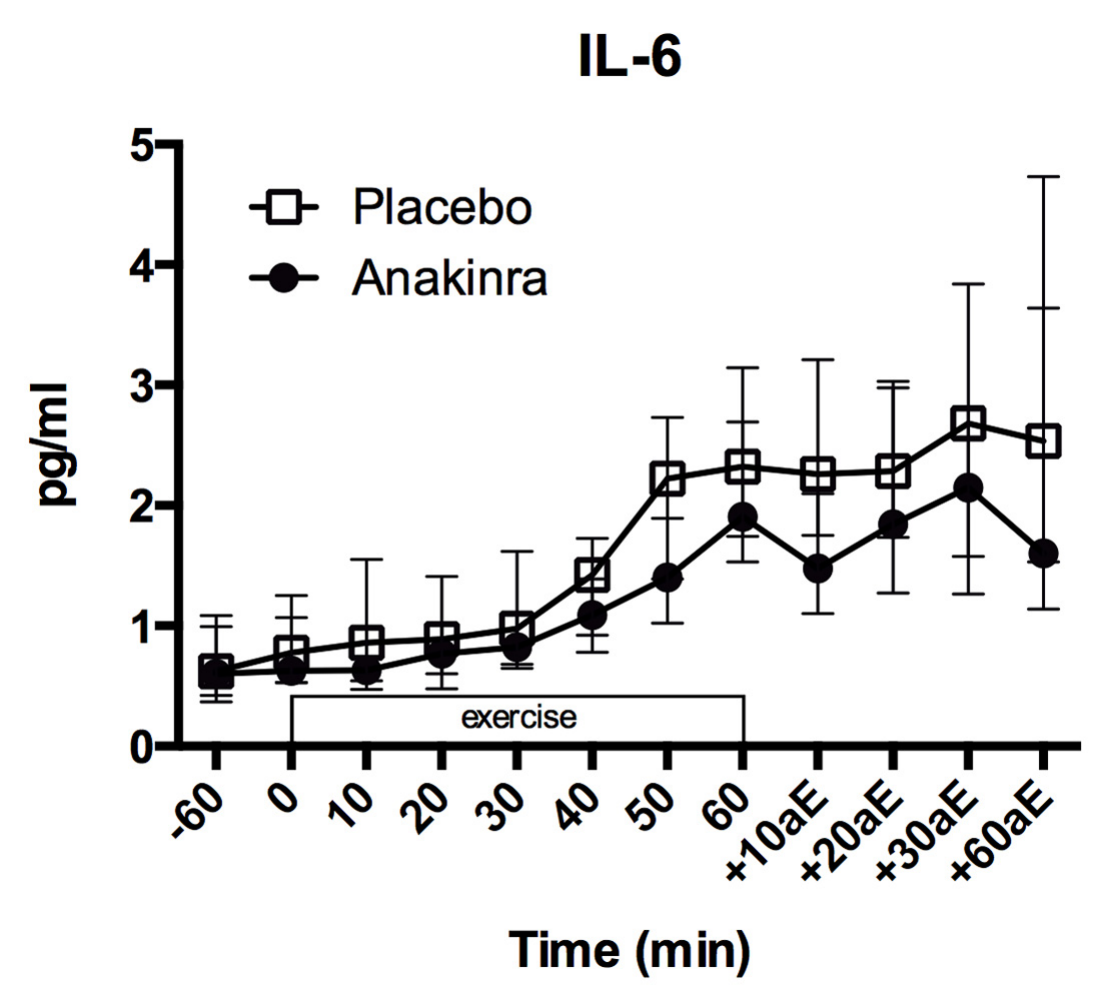
Plasma IL–6 levels. Plasma IL–6 levels before (-60 min.), during (0 to 60 min.) and after (+10aE to +60aE min.) exercise. Data represent the median and interquartile range, n = 17 subjects. aE = after exercise.

Plasma levels of IL–6 increased 2–3 fold from beginning to the end of exercise (Fig 2; placebo: 0.78 [0.55, 1.14] pg/ml to 2.32 [1.75, 3.11] pg/ml; anakinra: 0.63 [0.56, 0.95] pg/ml to 1.91 [1.54, 2.48] pg/ml) following a quadratic trend (p < 0.001). There was no difference in the increase of IL–6 levels over time between treatment groups (quadratic interaction: p = 0.189; linear Interaction: p = 0.595) and no overall difference in absolute IL–6 levels between placebo and anakinra treatment (p = 0.172). Estimated values per time point and model summary are provided in Table A and Table B in S1 File, respectively.

### IL–8, hs-CRP, MCP–1

**Fig 3:**
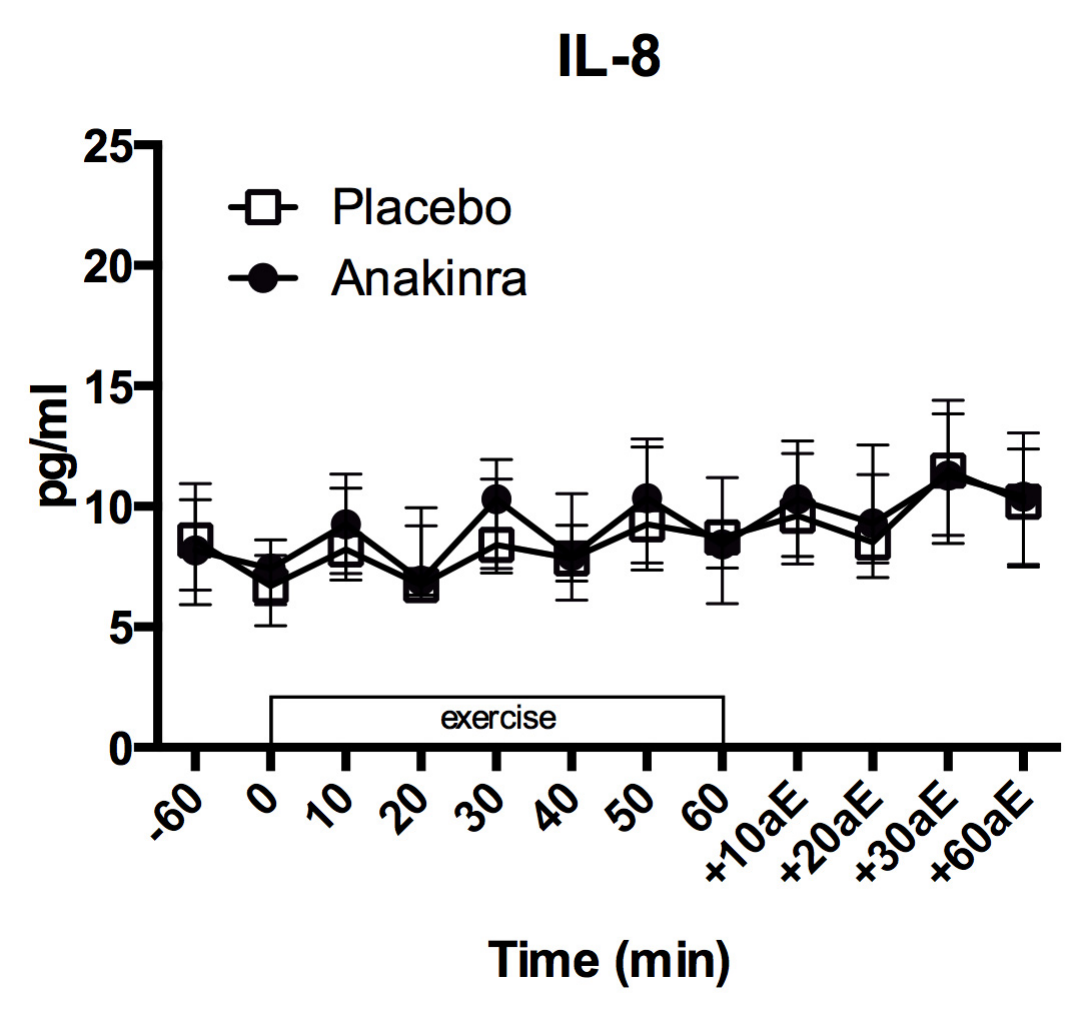
Plasma IL–8 levels. Plasma IL–8 levels before (-60 min.), during (0 to 60 min.) and after (+10aE to +60aE min.) exercise. Data represent the median and interquartile range, n = 17subjects. aE = after exercise.

Plasma levels of IL–8 slightly increased from beginning to the end of exercise (Fig 3; placebo: 6.70 [5.96, 7.38] pg/ml to 8.71 [6.31, 11.13] pg/ml; anakinra (7.54 [5.91, 9.09] pg/ml to 8.43 [7.35, 9.30] pg/ml) following a linear trend (p = <0.001). There was no difference in the increase of IL–8 levels over time between treatment groups (linear interaction p = 0.998; quadratic interaction p = 0.502) and no overall difference in absolute IL–8 levels between placebo and anakinra treatment (p = 0.743). Estimated values per time point and model summary and are provided in Table C and Table D in S1 File, respectively.

**Fig 4:**
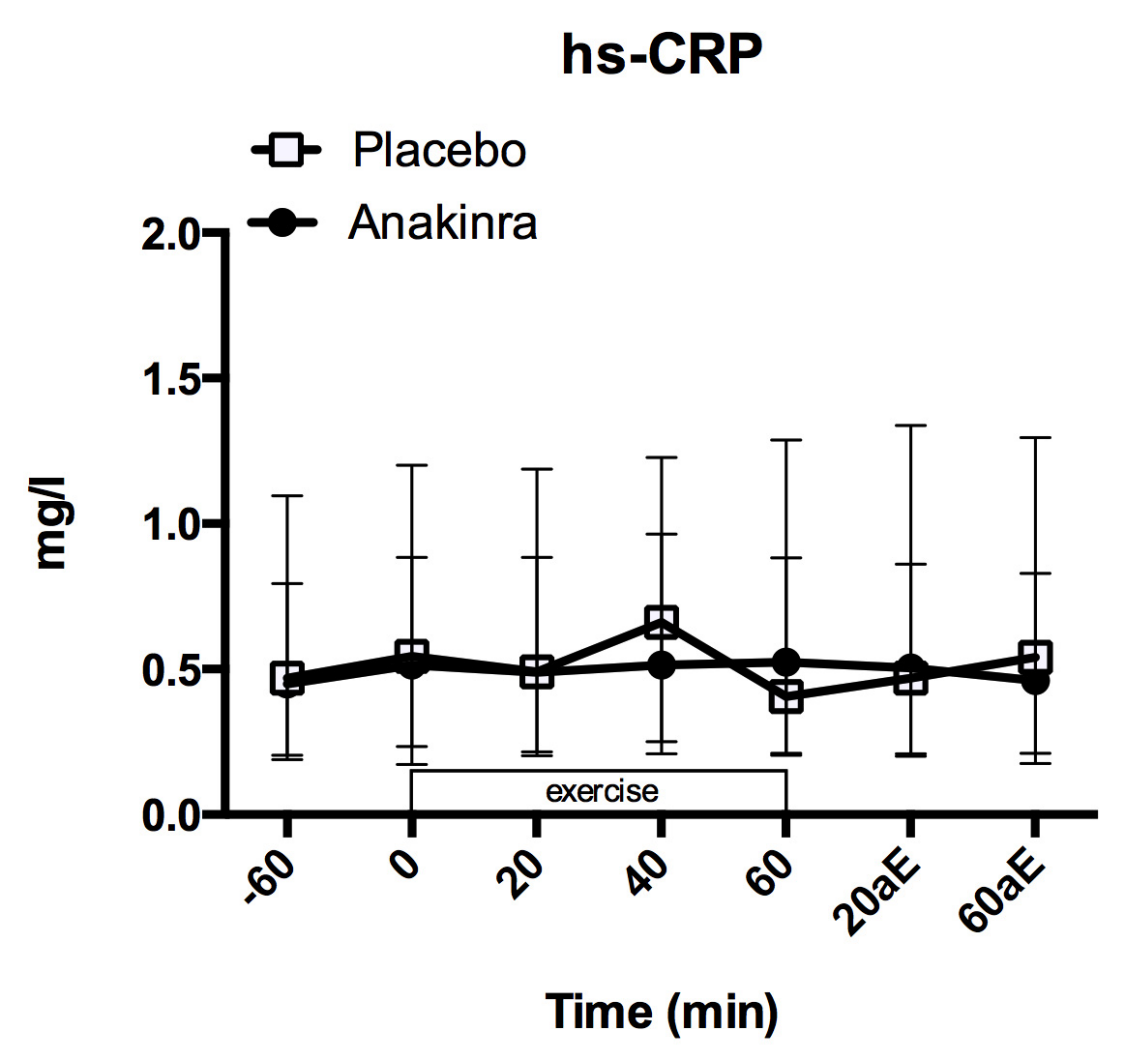
Plasma hs-CRP levels. Plasma hs-CRP levels before (-60 min.), during (0 to 60 min.) and after (+10aE to +60aE min.) exercise. Data represent the median and interquartile range, n = 17 subjects. aE = after exercise.

Hs-CRP did not change over time during exercise in both treatment groups (Fig 4; placebo: 0.55 [0.24, 0.85] mg/l to 0.41 [0.23, 0.85] mg/l; anakinra: 0.52 [0.20, 1.12] mg/l to 0.53 [0.22, 1.22] mg/l). Summary of the model is given in Table E in S1 File.

**Fig 5:**
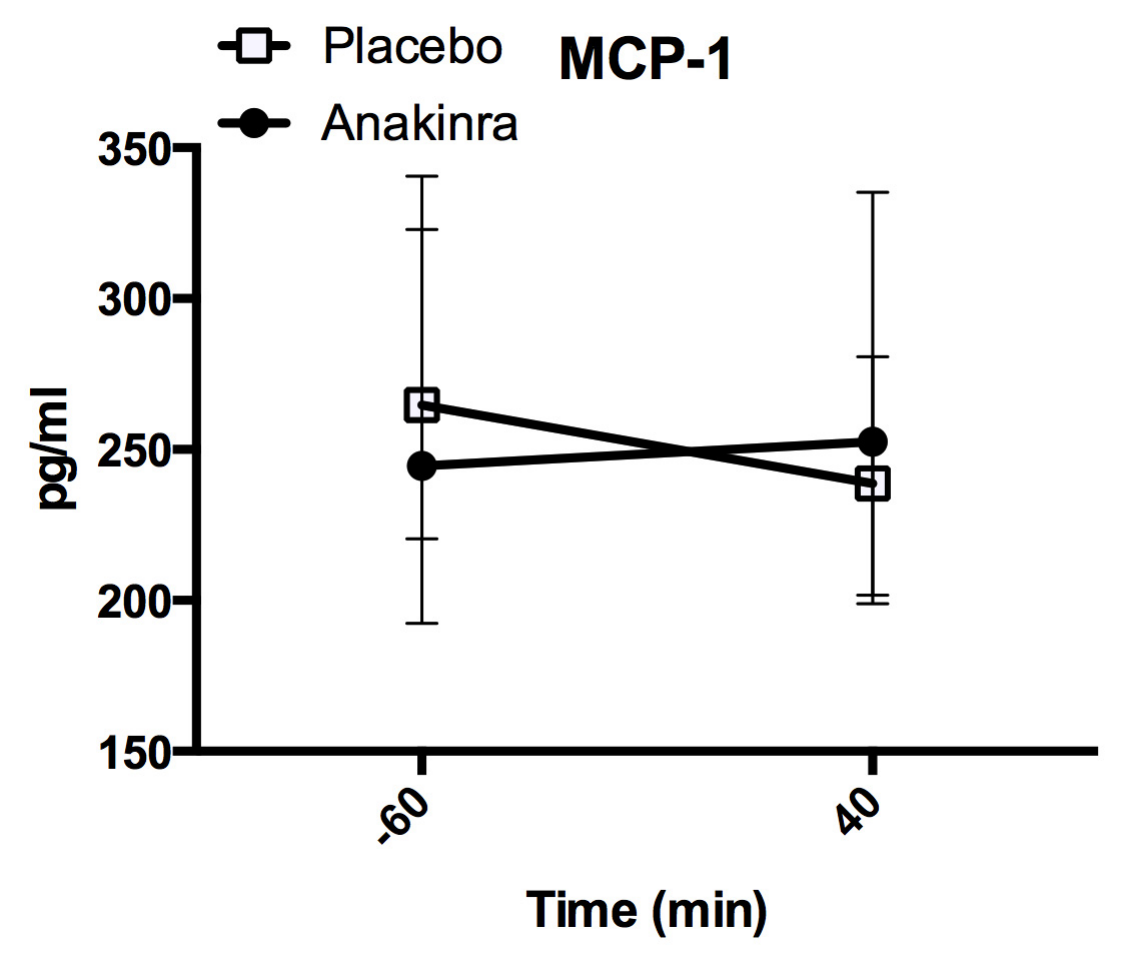
Plasma MCP–1 levels. Plasma MCP–1 levels before (-60 min.) and during (40 min.) exercise. Data represent the median and interquartile range, n = 17 subjects. aE = after exercise.

MCP–1, measured at 60 min. before and at 40 min. during exercise, decreased during exercise (Fig 5; placebo: 264.68 [224.63, 303.99] pg/ml to 238.75 [201.90, 278.36] pg/ml; anakinra 244.61 [201.84, 335.73] pg/ml to 252.62 [223.63, 327.62] pg/ml). There was no difference in the decrease of MCP–1 levels between treatment groups (p = 0.52) and no overall difference in absolute levels between placebo and anakinra (-60 min.: p = 0.970; 40 min.: p = 0.500).

### Glucose

**Fig 6:**
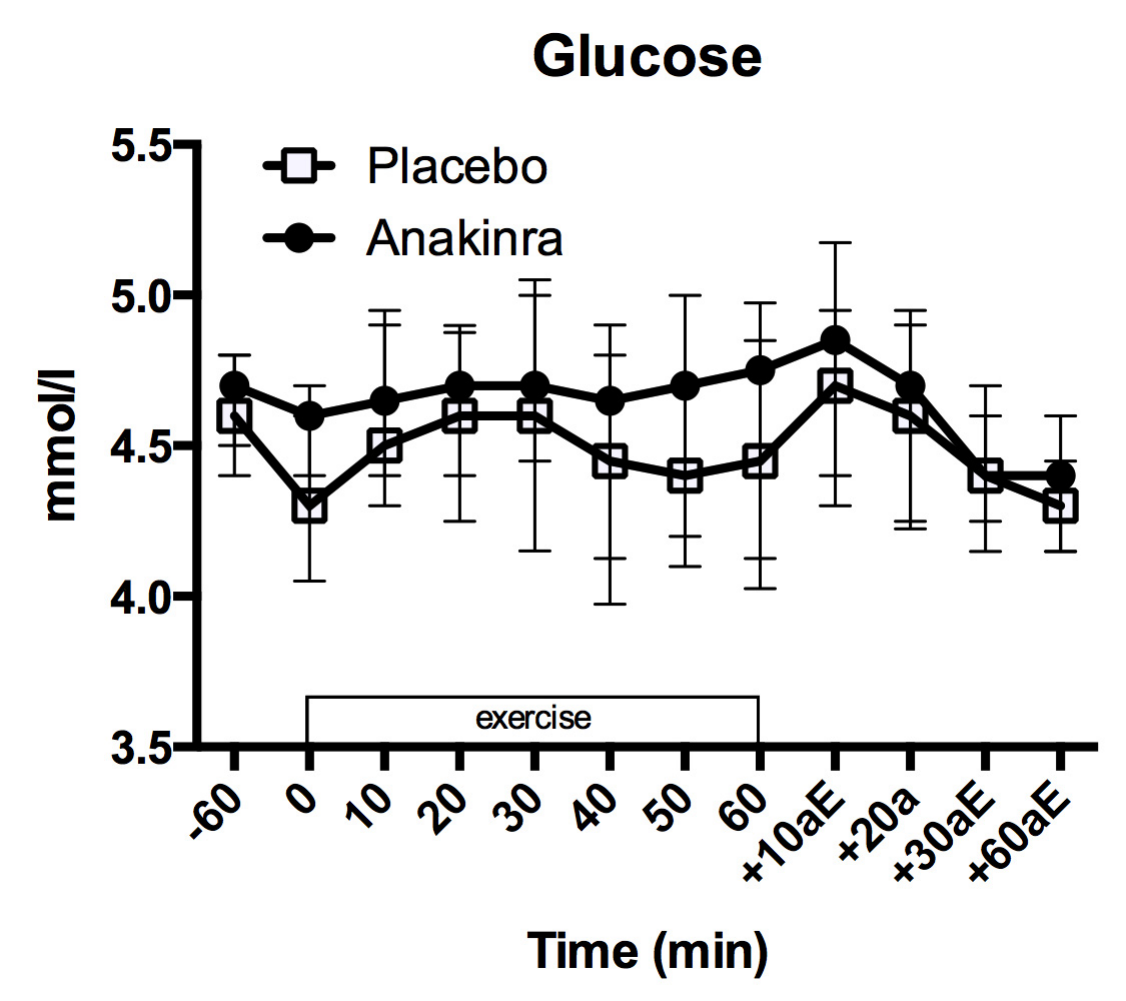
Plasma glucose levels. Plasma glucose levels before (-60 min.), during (0 to 60 min.) and after (+10aE to +60aE min.) exercise. Data represent the median and interquartile range, n = 17 subjects. aE = after exercise.

There was a significant decrease in glucose levels from injection time to exercise begin (Fig 6; placebo: 4.60 [4.40, 4.80] mmol/l to 4.30 [4.10, 4.60] mmol/l; anakinra: 4.70 [4.60, 4.80] mmol/l to 4.60 [4.40, 4.70] mmol/l; p = 0.038). The change in glucose values over time did not differ among the treatments (p = 0.466). Interestingly, glucose levels in the anakinra treated group were overall slightly higher compared to the placebo treated group (p = 0.06).

During exercise, glucose levels increased slightly (Fig 6; placebo: 4.30 [4.10, 4.60] mmol/l to 4.45 [4.07, 4.75] mmol/l; anakinra: 4.60 [4.40, 4.70] mmol/l to 4.75 [4.18, 4.93] mmol/l), following a quadratic trend (p <0.001). There was no difference in the increase of glucose levels over time between treatment groups (quadratic interaction p = 0.792; linear interaction p = 0.758) and, in contrast to the pre-exercise values, no difference in absolute values at any of the measured time points between placebo and anakinra (all p >0.11). Model summary is provided in Table F in S1 File.

### Cortisol

**Fig 7:**
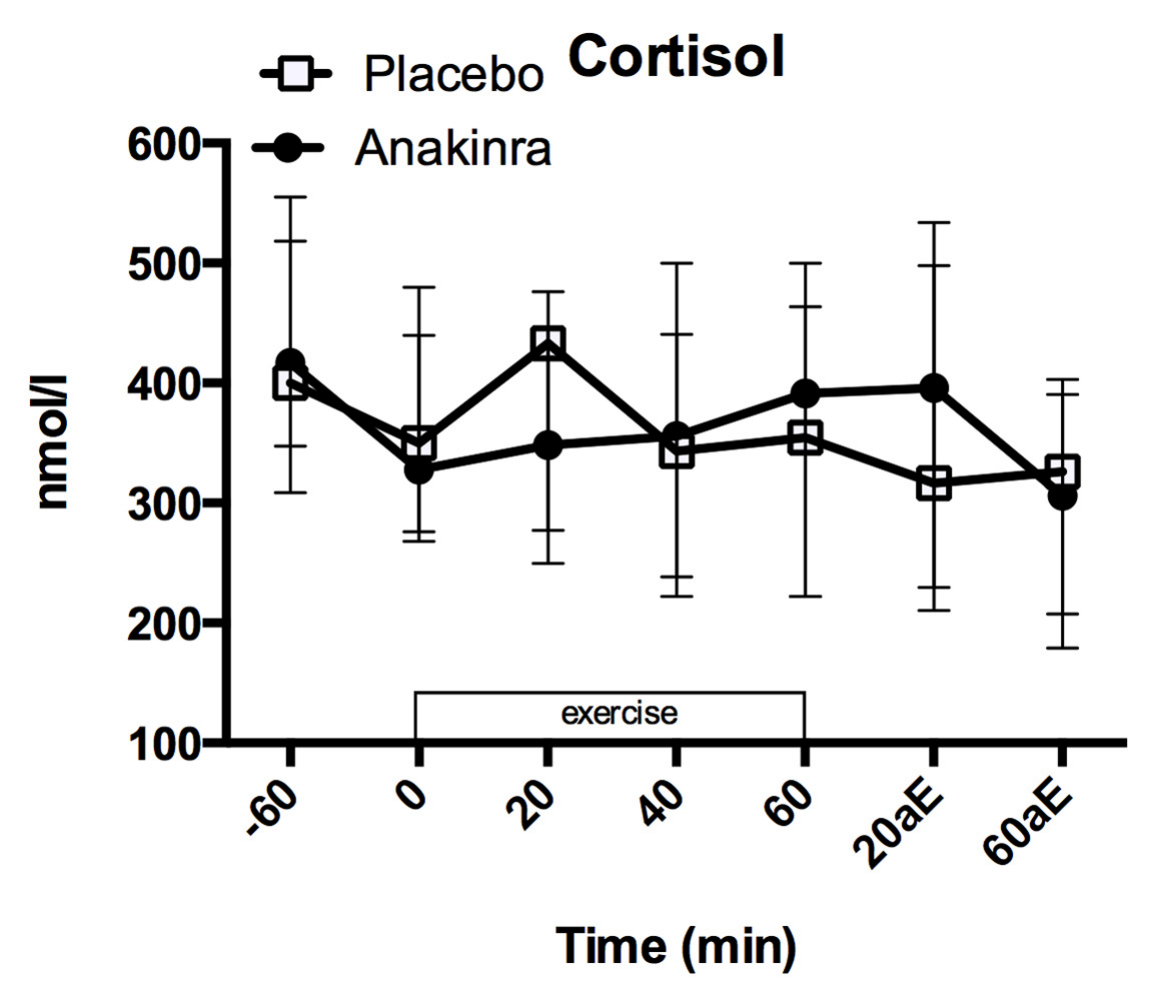
Plasma cortisol levels. Plasma cortisol levels before (-60 min.), during (0 to 60 min.) and after (+20aE, and+60aE min.) exercise (b). Data represent the median and interquartile range, n = 17 subjects. aE = after exercise.

There was a significant decrease in cortisol levels from injection time to exercise begin (Fig 7; placebo: 400 [320, 527] nmol/l to 350 [287, 441] nmol/l; anakinra 417 [349, 516] nmol/l to 328 [274, 428] nmol/l; p = 0.019). While the change from injection time until the begin of exercise was more pronounced in the anakinra treated subjects (placebo: -19 [-107.5, 22.5] nmol/l; anakinra: -87 [-110.5, -37.5] nmol/l) there was no significant decrease of cortisol levels over time (p = 0.436) and no overall difference in absolute cortisol levels between placebo and anakinra treatment (p = 0.902).

During exercise, cortisol levels did not change over time (Fig 7; placebo: 350.00 [287.00, 441.00] nmol/l to 354.50 [228.00, 450.50] nmol/l; anakinra: 328.00 [274.00, 428.00] nmol/l to 391.50 [222.75, 496.00] nmol/l) and did not differ between treatments overall (p = 0.912), or at any of the time points measured (all p > 0.32). Model summary is provided in Table G in S1 File.

### Creatine kinase

**Fig 8:**
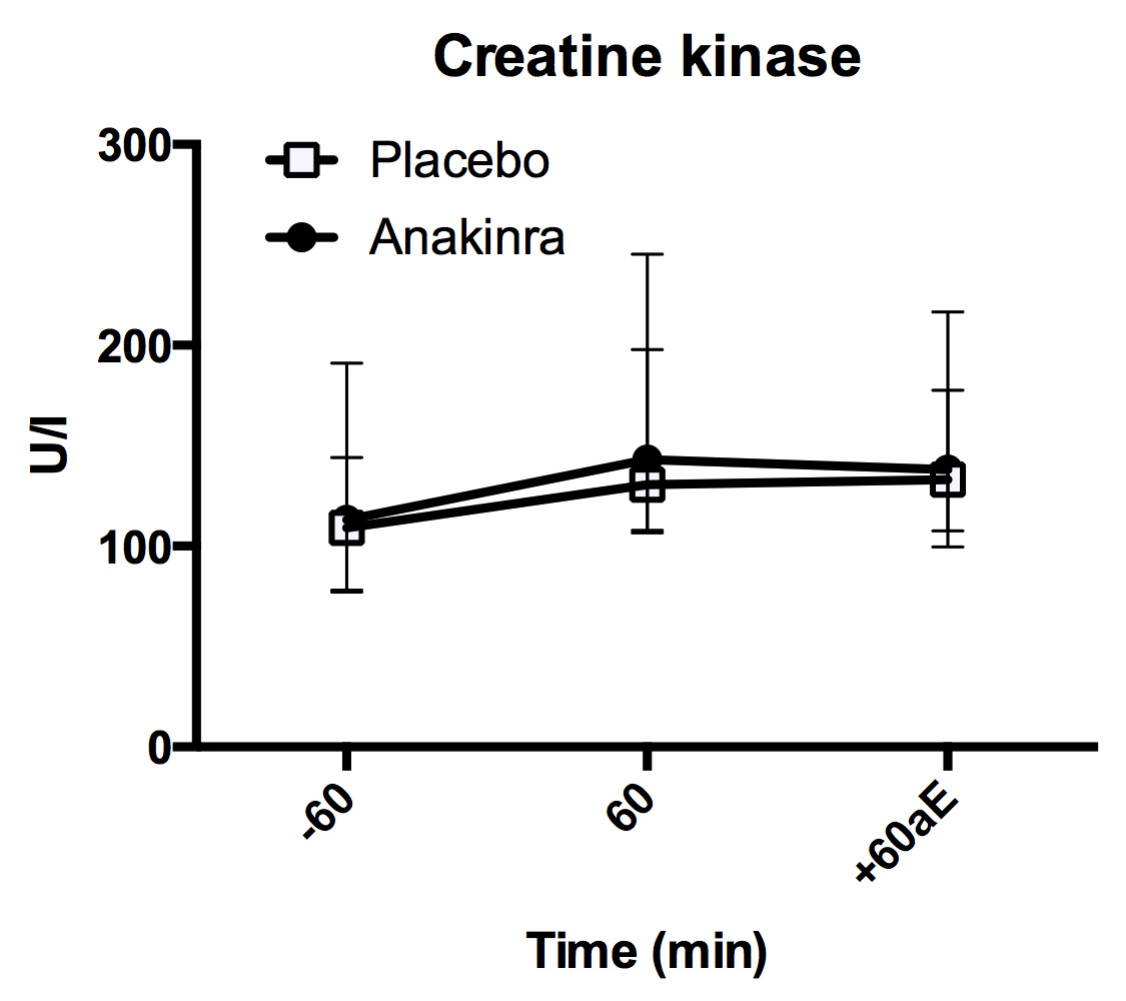
Plasma creatine kinase levels. Plasma creatine kinase levels before (-60 min.), during (60 min.) and after (+60aE min.) exercise. Data represent the median and interquartile range, n = 17 subjects. aE = after exercise.

Creatine kinase levels, measured 60 minutes before (-60 min.), at the end of exercise (60 min.) and 60 min. after exercise (60 min. aE), increased during exercise (Fig 8; placebo: 109.0 [79.0, 142.0] U/l to 130.5 [109.5, 189.25] U/l; anakinra 113.0 [81.0, 176.0] U/l to 143.0 [111.25, 227.25 U/l; p = <0.001). There was no difference in the increase over time between the treatment groups (p = 0.912) and no difference in absolute levels between placebo and anakinra treatment (p = 0.637). Model summary is provided in Table H in S1 File.

### Fatigue, processing and emotional tests

There was no difference in fatigue measurements between placebo and anakinra treatment (placebo: 25.5 [21.5, 30.05] points to 23 [21.0, 31.5] points; anakinra: 24.0 [21.0, 32.0] points to 25.5 [22.5, 31.5] points). Further, statistical analysis revealed a significant interaction of the treatment sequence (p = 0.05). Model summary is provided in Table I in S1 File.

There was no difference in the depression indices (placebo: 0 [0, 2] to 0 [0, 1.2] points; anakinra: 0 [0, 1.5] to 0 [0, 2]) before and after exercise and no difference between placebo and anakinra treatment. Interestingly, measurements were lower in the second study visit of each subject, independently of the treatment received. Model summary is provided in Table J in S1 File.

There were also no changes in processing and emotional tests (not shown).

### Biological activity of injected Anakinra

**Fig 9:**
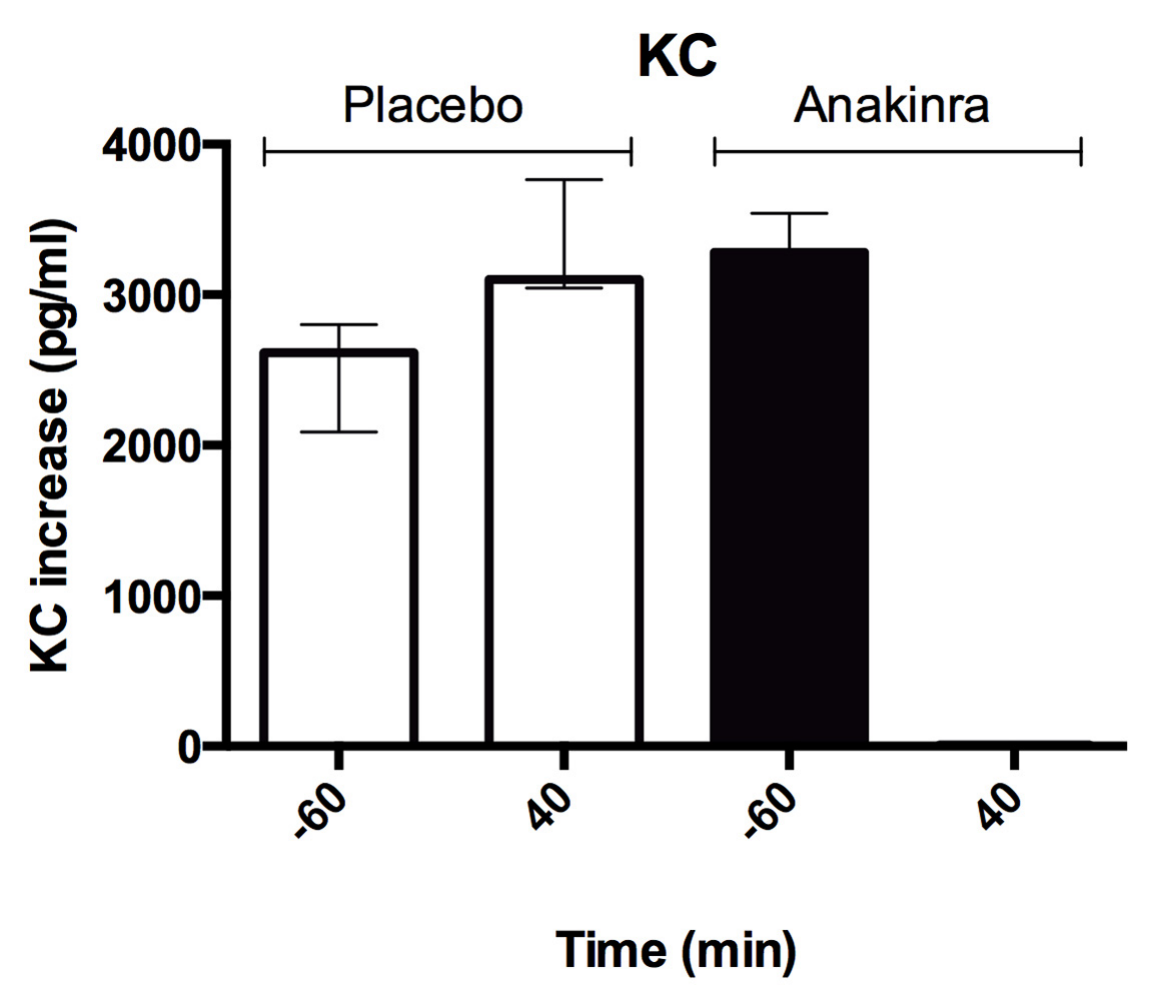
KC inhibition by anakinra-serum. Increase in KC concentration in supernatants of MIN6 cells exposed to IL–1β compared to solvent alone in the presence of serum from participant 6 before (-60 min.) and after (40 min.) placebo or anakinra administration. Data represent the median and interquartile range, n = 3wells/condition. KC = keratinocyte-derived chemokine.

Serum levels of IL-1Ra increased following injections of anakinra, along the randomization protocol (Table 2). To investigate whether the batch of injected anakinra led to biologically active inhibition of IL–1, serum of a subject was tested *in vitro* on the pancreatic β-cell line MIN6. As a read out, we used IL–1β induced KC, the rodent homologous of IL–8. IL–1β strongly induced KC secretion in the presence of placebo-serum but not in the presence of anakinra-serum (Fig 9).

**Table 2:** Serum interleukin–1 receptor antagonist levels. Serum interleukin–1 receptor antagonist levels (pg/ml) before (-60 min.) and after (40 min.) placebo or anakinra administration. P = participant number.

	Placebo		Anakinra	
P	-60min	40min	-60min	40min
1	328.1	363.6	321.1	25842.5
2	326.6	288.4	360.9	24677.8
3	638.6	579.6	496.5	25780.5
4	339.1	291.5	220.1	20263.2
5	250.7	297.1	275.2	27390.5
6	321.0	364.3	1154.0	24124.1
7	267.4	357.9	254.5	24338.5
8	423.9	363.3	219.2	25185.1
10	232.9	371.5	222.2	27815.5
13	290.9	315.4	251.3	26980.4
14	294.7	387.0	325.9	19689.2
15	294.7	351.7	254.8	25932.2
16	532.4	801.1	456.3	20687.9
17	283.8	369.3	275.2	22316.0
19	209.8	340.0	265.4	20442.1
20	241.6	257.3	260.3	19574.7
21	315.9	468.9	321.0	22807.9

## Discussion

The aim of this study was to explore the regulation of muscle-derived IL–6 during exercise and particularly, whether it is regulated via the IL–1 system. Consistent with previous findings [2], plasma IL–6 levels increased significantly during exercise. There was no difference in IL–6 levels after administration of the IL–1 receptor antagonist anakinra and placebo. Furthermore, the IL–1 responsive inflammatory markers IL–8, hsCRP and MCP–1 remained largely unaffected by exercise and anakinra. Therefore, our data support the concept that the release of IL–6 during exercise is a physiological response of the muscle and that it is not regulated by the pro-inflammatory IL–1 system. This finding is crucial in the context of evolving therapies with IL–1 antagonists in patients with type 2 diabetes. IL–1 is a strong inducer of autoinflammatoy processes leading to β-cell death and subsequently diabetes mellitus. It has been shown that IL–1 up regulates IL–6 in vitro [2]. In accordance, IL–1 antagonism decreases levels of IL–6 in chronic inflammatory disease [14]. But compared to IL–1, the role of IL–6 in the pathogenesis of diabetes is controversial. Studies indicating that IL–6 is associated with insulin resistance [5] are challenged by several findings showing that IL–6 actually has insulin-sensitizing effects [3] and that blocking of IL–6 may induce insulin resistance [15]. Moreover, IL–6 mediates insulin secretion during exercise by increasing secretion of glucagon-like peptide 1 [4]. Due to this beneficial effect of IL–6 on glucose metabolism during exercise, we note that there was no evidence of detrimental impact of the IL–1 receptor antagonist on exercise-induced increase of IL–6. Thus, we hypothesize that treatment strategies with IL–1 antagonists will not abolish the beneficial effect of exercise induced IL–6 on glucose metabolism.

The exercise load in our study, compared to other studies [16] is relatively mild, accounting for the only mildly elevated IL–6 levels we observed and limiting our study. Nevertheless, it was our aim to study the effect of muscle-derived IL–6 levels during a physiological setting, avoiding high-intensity activity with subsequent muscle damage. Indeed, the treadmill exercise at our target intensity level of 75% VO2max induced a significant increase in IL–6 levels without affecting pro-inflammatory parameters (hs-CRP, MCP–1, IL–8), which we found to be the optimal setting to study the effect of physiological, muscle-derived IL–6.

IL–1β has been shown to stimulate the release of adrenocorticotropic hormone [11]. While cortisol levels remained stable during exercise, we observed a decrease right after injection of either IL-1Ra or placebo and before physical activity had started. While both groups had a decrease in median cortisol levels, the reduction was more pronounced after treatment with IL-1Ra. It could be that IL-1Ra slightly decreases serum cortisol levels. It will take further investigation to specifically test this hypothesis.

Current literature suggests that IL-1ß is detrimental in the setting of type 2 diabetes and antagonizing it leads to an improvement in glucose homeostasis. In contrast, very low concentrations of IL-1ß promotes insulin secretion [17] possibly explaining that glucose levels were higher during the hour prior to exercise in IL-1Ra compared to placebo treated subjects.

In conclusion, antagonizing IL–1 does not seem to undermine the potential beneficial effect of exercise induced acute IL–6. This finding is important in the context of evolving therapies with IL–1 antagonists in patients with type 2 diabetes. Furthermore, we hypothesize that interleukin–1 receptor antagonist may have a favorable effect in some conditions linked to cortisol overproduction.

## Supporting Information

S1 File Estimated Least Square means (LS means) for log(IL6) as estimated following a linear mixed effects model (Table A). Estimated coefficients and standard errors for modeling log(IL6) measured during 60 minutes of exercise (Table B). Estimated Least Square means (LS-means) contrasts for log(IL8) as estimated following a linear mixed effects model (Table C). Estimated coefficients and standard errors for modeling log(IL8) measured during 60 minutes of exercise (Table D). Estimated coefficients and standard errors for modeling log(hsCRP) measured during 60 minutes of exercise (Table E). Estimated coefficients and standard errors for modeling Glucose (mmol/l) measured during 60 minutes of exercise (Table F). Estimated coefficients and standard errors for modeling log(Cortisol) measured during 60 minutes of exercise (Table G). Estimated coefficients and standard errors for modeling log(Creatine Kinase) measured during 60 minutes pre-exercise, and at the end of 60 minutes of exercise (Table H). Estimated coefficients and standard errors for modeling Fatigue before and after a 60 minutes exercise (Table I). Estimated coefficients and standard errors for modeling BDI measurements after a 60 minutes exercise (Table J).
(DOCX)

S2 File Consort 2010 Checklist. (DOC)

S3 File Study protocol. (DOC)

## References

[1] Eckardt K, Gorgens SW, Raschke S, Eckel J. Myokines in insulin resistance and type 2 diabetes. Diabetologia. 2014;57(6):1087–99. 10.1007/s00125-014-3224-x 24676645

[2] Febbraio MA, Pedersen BK. Muscle-derived interleukin–6: mechanisms for activation and possible biological roles. FASEB J. 2002;16(11):1335–47. 12205025 10.1096/fj.01-0876rev

[3] Benrick A, Wallenius V, Asterholm IW. Interleukin–6 mediates exercise-induced increase in insulin sensitivity in mice. Exp Physiol. 2012;97(11):1224–35. 10.1113/expphysiol.2012.065508 22523382

[4] Ellingsgaard H, Hauselmann I, Schuler B, Habib AM, Baggio LL, Meier DT, et al Interleukin–6 enhances insulin secretion by increasing glucagon-like peptide–1 secretion from L cells and alpha cells. Nat Med. 2011;17(11):1481–9. 10.1038/nm.2513 22037645 PMC4286294

[5] Pedersen BK, Febbraio MA. Muscles, exercise and obesity: skeletal muscle as a secretory organ. Nat Rev Endocrinol. 2012;8(8):457–65. 10.1038/nrendo.2012.49 22473333

[6] Stienstra R, Joosten LA, Koenen T, van Tits B, van Diepen JA, van den Berg SA, et al The inflammasome-mediated caspase–1 activation controls adipocyte differentiation and insulin sensitivity. Cell Metab. 2010;12(6):593–605. 10.1016/j.cmet.2010.11.011 21109192 PMC3683568

[7] Larsen CM, Faulenbach M, Vaag A, Volund A, Ehses JA, Seifert B, et al Interleukin-1-receptor antagonist in type 2 diabetes mellitus. N Engl J Med. 2007;356(15):1517–26. 17429083 10.1056/NEJMoa065213

[8] Sloan-Lancaster J, Abu-Raddad E, Polzer J, Miller JW, Scherer JC, De Gaetano A, et al Double-blind, randomized study evaluating the glycemic and anti-inflammatory effects of subcutaneous LY2189102, a neutralizing IL-1beta antibody, in patients with type 2 diabetes. Diabetes Care. 2013;36(8):2239–46. 10.2337/dc12-1835 23514733 PMC3714510

[9] Whitham M, Chan MH, Pal M, Matthews VB, Prelovsek O, Lunke S, et al Contraction-induced interleukin–6 gene transcription in skeletal muscle is regulated by c-Jun terminal kinase/activator protein–1. J Biol Chem. 2012;287(14):10771–9. 10.1074/jbc.M111.310581 22351769 PMC3322851

[10] Cavelti-Weder C, Furrer R, Keller C, Babians-Brunner A, Solinger AM, Gast H, et al Inhibition of IL-1beta improves fatigue in type 2 diabetes. Diabetes Care. 2011;34(10):e158 10.2337/dc11-1196 21949230 PMC3177746

[11] Cambronero JC, Rivas FJ, Borrell J, Guaza C. Interleukin-1-beta induces pituitary adrenocorticotropin secretion: evidence for glucocorticoid modulation. Neuroendocrinology. 1992;55(6):648–54. 1321354 10.1159/000126184

[12] Penner IK, Bechtel N, Raselli C, Stocklin M, Opwis K, Kappos L, et al Fatigue in multiple sclerosis: relation to depression, physical impairment, personality and action control. Mult Scler. 2007;13(9):1161–7. 17967844 10.1177/1352458507079267

[13] Benedict RH, Duquin JA, Jurgensen S, Rudick RA, Feitcher J, Munschauer FE, et al Repeated assessment of neuropsychological deficits in multiple sclerosis using the Symbol Digit Modalities Test and the MS Neuropsychological Screening Questionnaire. Mult Scler. 2008;14(7):940–6. 10.1177/1352458508090923 18573822

[14] Hung AM, Ellis CD, Shintani A, Booker C, Ikizler TA. IL-1beta receptor antagonist reduces inflammation in hemodialysis patients. J Am Soc Nephrol. 2011;22(3):437–42. 10.1681/ASN.2010070760 21310819 PMC3060437

[15] Wallenius V, Wallenius K, Ahren B, Rudling M, Carlsten H, Dickson SL, et al Interleukin-6-deficient mice develop mature-onset obesity. Nat Med. 2002;8(1):75–9. 11786910 10.1038/nm0102-75

[16] Pedersen BK, Hoffman-Goetz L. Exercise and the immune system: regulation, integration, and adaptation. Physiol Rev. 2000;80(3):1055–81. 10893431 10.1152/physrev.2000.80.3.1055

[17] Maedler K, Schumann DM, Sauter N, Ellingsgaard H, Bosco D, Baertschiger R, et al Low concentration of interleukin-1beta induces FLICE-inhibitory protein-mediated beta-cell proliferation in human pancreatic islets. Diabetes. 2006;55(10):2713–22. 17003335 10.2337/db05-1430

