# Supplementary material for: Muscle-Derived IL-6 Is Not Regulated by IL-1 during Exercise. A Double Blind, Placebo-Controlled, Randomized Crossover Study
Source: PLoS One. 2015 Oct 8;10(10):e0139662. doi: 10.1371/journal.pone.0139662 (PMC4597979; doi:10.1371/journal.pone.0139662)
Supplement: S1 File — Estimated coefficients and standard errors for modeling log(IL6) measured during 60 minutes of exercise (Table B). Estimated Least Square means (LS-means) contrasts for log(IL8) as estimated following a linear mixed effects model (Table C). Estimated coefficients and standard errors for modeling log(IL8) measured during 60 minutes of exercise (Table D). Estimated coefficients and standard errors for modeling log(hsCRP) measured during 60 minutes of exercise (Table E). Estimated coefficients and standard errors for modeling Glucose (mmol/l) measured during 60 minutes of exercise (Table F). Estimated coefficients and standard errors for modeling log(Cortisol) measured during 60 minutes of exercise (Table G). Estimated coefficients and standard errors for modeling log(Creatine Kinase) measured during 60 minutes pre-exercise, and at the end of 60 minutes of exercise (Table H). Estimated coefficients and standard errors for modeling Fatigue before and after a 60 minutes exercise (Table I). Estimated coefficients and standard errors for modeling BDI measurements after a 60 minutes exercise (Table J). (DOCX) [file pone.0139662.s001.docx]

| Time (min) | Treatment | LS mean | CI | p |
| --- | --- | --- | --- | --- |
|  | P | -0.21 | [-0.52, 0.11] |  |
| 0 | V | -0.23 | [-0.54, 0.09] | 0.889 |
|  | P | -0.04 | [-0.35, 0.28] |  |
| 10 | V | -0.29 | [-0.60, 0.03] | 0.069 |
|  | P | -0.07 | [-0.38, 0.25] |  |
| 20 | V | -0.20 | [-0.52, 0.11] | 0.324 |
|  | V | -0.13 | [-0.44, 0.19] |  |
| 30 | P | 0.07 | [-0.24, 0.39] | 0.143 |
|  | P | 0.31 | [-0.01, 0.63] |  |
| 40 | V | 0.15 | [-0.16, 0.46] | 0.244 |
|  | P | 0.67 | [0.36, 0.99] |  |
| 50 | V | 0.46 | [0.15, 0.78] | 0.124 |
|  | P | 0.88 | [0.57, 1.20] |  |
| 60 | V | 0.77 | [0.45, 1.09] | 0.409 |

**Table A in S1 File.** **Estimated Least Square means (LS means) for log(IL6) as estimated following a linear mixed effects model.** The model incorporated Period and Treatment as ﬁxed effects. Subject ID was included as a random factor. Within subject correlation along time was incorporated by including an auto-regressive (AR1) function. P-values test the difference in log(IL-6) between treatment arms at each time-point.

**Table B in S1 File. Estimated coefﬁcients and standard errors for modeling log(IL6) measured during 60 minutes of exercise.** Coming from a crossover design, period represents the ﬁrst or measurement period of each subject. A mixed-effects model with subject- ID as random effect, and an auto-regressive (order 1) correlation structure was ﬁt to the data.

| Variable | Value | Std.Error | DF | t-value | p-value |
| --- | --- | --- | --- | --- | --- |
| (Intercept) | -0.197 | 0.146 | 210.000 | -1.349 | 0.179 |
| Period (2nd) | 0.087 | 0.092 | 210.000 | 0.949 | 0.344 |
| Time | -0.001 | 0.004 | 210.000 | -0.246 | 0.806 |
| *T ime*2 | 0.000 | 0.000 | 210.000 | 4.788 | 0.000 |
| Treatment (Verum) | -0.126 | 0.092 | 210.000 | -1.372 | 0.172 |

**Table C in S1 File.** **Estimated Least Square means (LS-means) contrasts for log(IL8) as estimated following a linear mixed effects model**. Contrasts shown as the difference (*time*0 - *timeminutes*), that is a negative estimate reﬂects an increase in IL-8 values. The model incorporated Period and Treatment as ﬁxed effects. Subect ID was included as a random factor. Within subject correlation along time was incorporated by including an auto-regressive (AR1) function. P-values test for a difference in log(IL-8) values between time-points within each treatment. At no time point was the difference between the treatment arms significant (all p > 0.45)

| contrast | Trt | estimate | SE | df | t.ratio | p.value |
| --- | --- | --- | --- | --- | --- | --- |
| 0 - 10 | P | -0.269 | 0.064 | 202.000 | -4.228 | *<*0.001 |
|  | V | -0.224 | 0.065 | 202.000 | -3.462 | 0.011 |
| 0 - 20 | P | -0.069 | 0.072 | 202.000 | -0.953 | 0.963 |
|  | V | -0.076 | 0.072 | 202.000 | -1.067 | 0.937 |
| 0 - 30 | P | -0.298 | 0.076 | 202.000 | -3.943 | 0.002 |
|  | V | -0.234 | 0.074 | 202.000 | -3.149 | 0.031 |
| 0 - 40 | P | -0.171 | 0.076 | 202.000 | -2.240 | 0.279 |
|  | V | -0.125 | 0.075 | 202.000 | -1.661 | 0.643 |
| 0 - 50 | P | -0.348 | 0.075 | 202.000 | -4.625 | *<*0.001 |
|  | V | -0.293 | 0.075 | 202.000 | -3.897 | 0.003 |
| 0 - 60 | P | -0.227 | 0.077 | 202.000 | -2.963 | 0.052 |
|  | V | -0.250 | 0.076 | 202.000 | -3.272 | 0.021 |

**Table D in S1 File. Estimated coefﬁcients and standard errors for modeling log(IL8) measured during 60 minutes of exercise.** Coming from a crossover design, period represents the ﬁrst or measurement period of each subject. A mixed-effects model with subject-ID as random effect, and an auto-regressive (order 1) correlation structure, after removal of non-signiﬁcant interactions, was ﬁt to the data.

| Variable | Value | Std.Error | DF | t-value | p-value |
| --- | --- | --- | --- | --- | --- |
| (Intercept) | 2.055 | 0.119 | 213.000 | 17.232 | 0.000 |
| Period (2nd) | 0.011 | 0.032 | 213.000 | 0.339 | 0.735 |
| Time | 0.004 | 0.001 | 213.000 | 4.510 | 0.000 |
| Treatment (Verum) | 0.011 | 0.032 | 213.000 | 0.328 | 0.743 |

**Table E in S1 File. Estimated coefﬁcients and standard errors for modeling log(hsCRP) measured during 60 minutes of exercise.** Coming from a crossover design, period rep- resents the ﬁrst or measurement period of each subject. A mixed-effects model with subject-ID as random effect, and an auto-regressive (order 1) correlation structure was ﬁt to the data.

| Variable | Value | Std.Error | DF | t-value | p-value |
| --- | --- | --- | --- | --- | --- |
| (Intercept) | -0.754 | 0.275 | 106.000 | -2.736 | 0.007 |
| Period (2nd) | 0.030 | 0.142 | 106.000 | 0.210 | 0.834 |
| TimeC | 0.000 | 0.000 | 106.000 | 0.014 | 0.989 |
| Treatment (Verum) | 0.139 | 0.142 | 106.000 | 0.981 | 0.329 |

| Variable | Value | Std.Error | DF | t-value | p-value |
| --- | --- | --- | --- | --- | --- |
| (Intercept) | 4.357 | 0.132 | 210.000 | 33.054 | 0.000 |
| Period (2nd) | 0.058 | 0.095 | 210.000 | 0.605 | 0.546 |
| Time | 0.013 | 0.004 | 210.000 | 3.547 | 0.000 |
| Time2 | -0.000 | 0.000 | 210.000 | -3.561 | 0.000 |
| Treatment (Verum) | 0.147 | 0.095 | 210.000 | 1.539 | 0.125 |

**Table F in S1 File. Estimated coefﬁcients and standard errors for modeling Glucose (mmol/l) measured during 60 minutes of exercise.** Coming from a crossover design, period represents the ﬁrst or measurement period of each subject. A mixed-effects model with subject-ID as random effect, and an auto-regressive (order 1) correlation structure was ﬁt to the data.

**Table G in S1 File. Estimated coefﬁcients and standard errors for modeling log(Cortisol) measured during 60 minutes of exercise.** Coming from a crossover design, period rep- resents the ﬁrst or measurement period of each subject. A mixed-effects model with subject-ID as random effect, and an auto-regressive (order 1) correlation structure was ﬁt to the data.

| Variable | Value | Std.Error | | DF | t-value | p-value | |
| --- | --- | --- | --- | --- | --- | --- | --- |
| (Intercept) | 5.868 | | 0.112 | 112.000 | 52.579 | | 0.000 |
| Period (2nd) | -0.026 | | 0.080 | 112.000 | -0.318 | | 0.751 |
| Time | -0.001 | | 0.001 | 112.000 | -1.426 | | 0.157 |
| Treatment (Verum) | -0.009 | | 0.080 | 112.000 | -0.110 | | 0.912 |

| Variable | Value | Std.Error | DF | t-value | p-value |
| --- | --- | --- | --- | --- | --- |
| (Intercept) | 4.909 | 0.178 | 46.000 | 27.648 | 0.000 |
| Period (2nd) | -0.115 | 0.068 | 46.000 | -1.677 | 0.100 |
| Time (60 min) | 0.277 | 0.068 | 46.000 | 4.044 | 0.000 |
| Treatment (Verum) | 0.033 | 0.068 | 46.000 | 0.476 | 0.637 |

**Table H in S1 File. Estimated coefﬁcients and standard errors for modeling log(Creatine Kinase) measured during 60 minutes pre-exercise, and at the end of 60 minutes of exercise.** Coming from a crossover design, period represents the ﬁrst or second measurement period of each subject. A mixed-effects model with subject-ID as random effect.

**Table I in S1 File.** **Estimated coefﬁcients and standard errors for modeling Fatigue before and after a 60 minutes exercise.** Coming from a crossover design, period represents the ﬁrst or second measurement period of each subject. A mixed-effects model with subject-ID as random effect.

| MotoVariable | Value | Std.Error | DF | t-value | p-value |
| --- | --- | --- | --- | --- | --- |
| (Intercept) | 7.615 | 5.048 | 15.000 | 1.508 | 0.152 |
| TOTpre | 0.581 | 0.122 | 12.000 | 4.780 | 0.000 |
| Period (2nd) | 5.337 | 3.663 | 12.000 | 1.457 | 0.171 |
| Treatment (Verum) | 6.502 | 3.692 | 12.000 | 1.761 | 0.104 |
| Period (2nd):Treatment (Verum) | -11.967 | 6.100 | 12.000 | -1.962 | 0.073 |

**Table J in S1 File.** **Estimated coefﬁcients and standard errors for modeling BDI measurements after a 60 minutes exercise.** Coming from a crossover design, period represents the ﬁrst or second measurement period of each subject. Measurements effects controlled for pre-exercise measurements. A mixed-effects model with subject-ID as random effect.

| Variable | Value | Std.Error | DF | t-value | p-value |
| --- | --- | --- | --- | --- | --- |
| (Intercept) | 0.502 | 0.181 | 16.000 | 2.769 | 0.014 |
| BDIpre | 0.368 | 0.051 | 12.000 | 7.230 | 0.000 |
| Period (2nd) | -0.245 | 0.108 | 12.000 | -2.271 | 0.042 |
| Treatment (Verum) | -0.022 | 0.109 | 12.000 | -0.200 | 0.845 |
