# Supplementary material for: Muscle-Derived IL-6 Is Not Regulated by IL-1 during Exercise. A Double Blind, Placebo-Controlled, Randomized Crossover Study
Source: PLoS One. 2015 Oct 8;10(10):e0139662. doi: 10.1371/journal.pone.0139662 (PMC4597979; doi:10.1371/journal.pone.0139662)
Supplement: S3 File — (DOC) [file pone.0139662.s003.doc]

APPROVAL OF CLINICAL TRIAL PROTOCOL

**Product:** Anakinra

**Protocol Title:** Role of IL-1 in the regulation of muscle derived IL-6 during exercise

**Study ID: 294/10**

**Protocol Number:** 11.0 Date 08.07.2011

Amendment 2 Date 08.07.2011

| Approved By: |  |  |  |
| --- | --- | --- | --- |
|  | Prof. Marc Donath  *(principal investigator & sponsor)* | Date: |  |
|  |  |  |  |

|  | Clinical TRIAL Protocol |
| --- | --- |
| Title: | **Role of IL-1 in the regulation of muscle derived IL-6 during exercise**  A Randomized, Double blind, Placebo-controlled, cross-over proof-of-concept study |
| Protocol Number: | 10.0 |
| Amendment: | 1 |
| Study Drug: | Anakinra |
| Sponsor/Principal Investigator: | Marc Y. Donath, MD, Professor Division of Endocrinology, Diabetes and Metabolism  Department of Internal Medicine University Hospital Basel, Switzerland +41 61 328 70 78 |
| Original Protocol Date: | May 10, 2010 |

STUDY SITE

The only study site will be at the University Hospital Basel, Switzerland.

Address:

Universitätsspital Basel
Petersgraben 4
4031 Basel
Schweiz
Telefon +41 61 265 25 25

STUDY PERSONNEL

| Prof. Marc Donath  *(principal investigator & sponsor)* |
| --- |
| Dr. Eleonora Seelig  *(investigator)* |
| Dr. Katharina Timper  *(investigator)* |
| Patrizia Zala  *(study nurse)*  PD Dr. Iris-Katharina Penner  *(collaborator for neuropsychological measures)*  Prof. Dr. Arno Schmidt-Trucksäss  *(substudy investigator)*  Dr. Mareike Cordes  *(substudy investigator)* |

# SYNOPSIS

| Protocol Title: | **Role of IL-1** **in the regulation of muscle derived IL-6 during exercise** |
| --- | --- |
| Sponsor/Investigator: | Marc Y. Donath, MD, Professor, Division of Endocrinology, Diabetes and Metabolism Department of Internal Medicine  University Hospital Basel, Switzerland |
| Protocol Number: | 10.0 |
| Amendment | 1 |
| Study Phase: | 2 |
| Study Drug: | Anakinra (Kineret) and Placebo |
| Study Population: | Healthy lean males |
| Objective: | Evaluate the regulation of muscle derived IL-6 during exercise and in particular whether it is regulated by the IL-1 system. |
| Program Hypothesis: | Muscle derived IL-6 is regulated by IL-1 vs. independently of the IL-1 system during an acute exercise bout |
| Study Design: | Placebo-controlled, double blind, cross-over, randomized, proof-of-concept |
| Sample Size: | 20 subjects |
| Estimated Start: | July, 2011 |
| Estimated Finish: | December, 2011 |
| No. of Sites: | 1 |
| Mean Study Duration for Subjects: | 36d  (screening visit included) |
| Eligibility Criteria: | Inclusion Criteria  Subjects may be included in the study if they meet all of the following criteria:   - male - non-smoking - apparently healthy - BMI >18 and <26kg/m2 - Age 20-50 years - Regular exercise including a minimum of two runs weekly of a total duration of > 2h - Willingness to use contraceptive measures adequate to prevent the subject’s partner from becoming pregnant during the study. Adequate contraceptive measures include hormonal methods used for two or more cycles prior to screening (e.g., oral contraceptive pills, contraceptive patch, or contraceptive vaginal ring), double barrier methods (e.g., contraceptive sponge, diaphragm used in conjunction with contraceptive foam or jelly, and condom used in conjunction with contraceptive foam or jelly), intrauterine methods (IUD), sterilization (e.g., tubal ligation or a monogamous relationship with a vasectomized partner), and abstinence. |
|  | Exclusion Criteria  Subjects will be excluded from the study if they meet any of the following criteria:   - Clinical signs of infection in the week before inclusion or history of infection during the last 3 months (CRP >5mg/l) - Impaired fasting glucose (fasting plasma glucose >5.5mmol/l) - Hematologic disease (leukocyte count < 1.5x10^9^/l, hemoglobin <11 g/dl, platelets <100 x 10^3^/ul) - Kidney disease (creatinine > 1.5 mg/dL for men and 1.4mg/dL for woman) - Liver disease (transaminases >2x upper normal range) - Heart disease - Pulmonary disease - Inflammatory disease - History of carcinoma - History of tuberculosis - Alcohol consumption >40g/d - Known allergy to Kineret - Current treatment with any drug in the week before inclusion, including vitamin supplementation (especially vitamin C and E) - Use of any investigational drug within 30 days prior to enrollment or within 5 half-lives of the investigational drug, whichever is longer - Subject refusing or unable to give written informed consent |
| Endpoints/ Outcome Measures: | ***Primary***:  The primary endpoint is change in exercise-stimulated IL-6 between group dosed with placebo and anakinra  Secondary:   - Change in glucose metabolism, insulin, glucagon and GLP-1 secretion due to any treatment. - Change of inflammatory markers (CRP, TNFα, IL-1Ra) due to any treatment. - Change of stress hormones (cortisol, growth hormone) due to any treatment. - Change of muscular enzymes (CK) due to any treatment. - Well-being during and after exercise including muscle soreness due to any treatment. - Activity Induced Fatigue (ACTIF) Scale before and after exercise - Depression - Cognition - Motor strength - Change of vascular function (CAVI, PWV and AVR) due to any treatment. |
| Safety Evaluations: | All recommendations outlined in the ICH Guidelines for Good Clinical Practice will be adhered to throughout this trial. The safety of the drug will be assessed by multiple subject assessments of vital signs, physical exams, clinical tests, and laboratory evaluations, and monitoring of adverse events (AEs). Concomitant medications will be assessed. |
| Biological Evaluations: | The following tests will be used to investigate how IL-6 is regulated (see Appendix 2 for time points):   - IL-6 - GLP -1 (active) - GIP - Glucagon - Insulin - Glucose - IL-1Ra - CRP - TNF-α - Cortisol - GH - CK |
| Sample Size: | The sample size (20 subjects) was based on clinical and practical considerations. |
| Statistical Considerations: | Subject demographics, medical history, and prior medications will be summarized by treatment group using descriptive statistics. All randomized subjects who receive at least one study treatment will be considered for analysis. Endpoints will be summarized descriptively and graphically by treatment and study visit. |

# TABLE OF CONTENTS

SYNOPSIS [5](#__RefHeading___Toc274753257)

TABLE OF CONTENTS [8](#__RefHeading___Toc274753258)

LIST OF TABLES [10](#__RefHeading___Toc274753259)

list of Figures [10](#__RefHeading___Toc274753260)

LIST OF APPENDICES [10](#__RefHeading___Toc274753261)

LIST OF ABBREVIATIONS [12](#__RefHeading___Toc274753262)

1.0 background [14](#__RefHeading___Toc274753263)

1.1 Overview [14](#__RefHeading___Toc274753264)

1.2 Disease Pathophysiology [14](#__RefHeading___Toc274753265)

1.3 Clinical Data [15](#__RefHeading___Toc274753266)

1.4 Anakinra/Kineret® [15](#__RefHeading___Toc274753267)

1.5 Study Rationale [15](#__RefHeading___Toc274753268)

2.0 OBJECTIVES [17](#__RefHeading___Toc274753269)

3.0 Study Design [18](#__RefHeading___Toc274753270)

3.1 Description of the Study [18](#__RefHeading___Toc274753271)

3.2 Rationale for Study Design and Dose Level [18](#__RefHeading___Toc274753272)

3.2.1 Rationale for Dose Level [18](#__RefHeading___Toc274753273)

3.2.1.1 Anakinra/Kineret® [18](#__RefHeading___Toc274753274)

3.2.2 Rationale for Study Design [19](#__RefHeading___Toc274753275)

3.3 Selection of Study Population [19](#__RefHeading___Toc274753276)

3.3.1 Subject Recruitment [19](#__RefHeading___Toc274753277)

3.3.2 Inclusion Criteria [19](#__RefHeading___Toc274753278)

3.3.3 Exclusion Criteria [19](#__RefHeading___Toc274753279)

3.4 Prior and Concomitant Therapy [20](#__RefHeading___Toc274753280)

3.5 Enrollment, Treatment Assignment, Blinding, and Unblinding [20](#__RefHeading___Toc274753281)

3.5.1 Enrollment [20](#__RefHeading___Toc274753282)

3.5.2 Treatment Assignment [20](#__RefHeading___Toc274753283)

3.5.3 Treatment Blinding [20](#__RefHeading___Toc274753284)

3.5.4 Treatment Unblinding [21](#__RefHeading___Toc274753285)

3.6 Treatment [21](#__RefHeading___Toc274753286)

3.6.1 Formulation of Anakinra/Kineret® [21](#__RefHeading___Toc274753287)

3.6.2 Labeling [22](#__RefHeading___Toc274753288)

3.6.3 Shipping of study drugs [22](#__RefHeading___Toc274753289)

3.6.4 Dosage, Preparation, Administration, and Storage [22](#__RefHeading___Toc274753290)

3.6.4.1 Dosage [22](#__RefHeading___Toc274753291)

3.6.4.2 Preparation [23](#__RefHeading___Toc274753292)

3.6.4.3 Administration [23](#__RefHeading___Toc274753293)

3.6.4.4 Storage and Handling [23](#__RefHeading___Toc274753294)

3.6.4.5 Drug Accountability [23](#__RefHeading___Toc274753295)

3.7 Selection of Doses Used in the Study [24](#__RefHeading___Toc274753296)

3.8 Outcome Measures [24](#__RefHeading___Toc274753297)

3.8.1 Primary Outcome Measure [24](#__RefHeading___Toc274753298)

3.8.2 Secondary Outcome Measures [24](#__RefHeading___Toc274753299)

3.8.3 Safety Outcome Measures [25](#__RefHeading___Toc274753300)

3.9 Subject costs and insurance [25](#__RefHeading___Toc274753301)

3.10 Subject Compensation [26](#__RefHeading___Toc274753302)

3.11 Financing [26](#__RefHeading___Toc274753303)

4.0 STUDY OPERATIONS and Evaluations [27](#__RefHeading___Toc274753304)

4.1 Reporting and Recording of Data [27](#__RefHeading___Toc274753305)

4.2 Study Assessments [27](#__RefHeading___Toc274753306)

4.2.1 Screening Assessments (Day -28 to -7) [27](#__RefHeading___Toc274753307)

4.2.2 Assessments for Treatment and Follow-up Period [28](#__RefHeading___Toc274753308)

4.2.2.1 Visit 1 (Day 0) [28](#__RefHeading___Toc274753309)

4.2.2.2 Visit 2 [Day 6 (± 3 Days)] [30](#__RefHeading___Toc274753310)

4.2.2.3 Visit 3 [Day 12 (±9 Days)] [32](#__RefHeading___Toc274753311)

4.2.2.4 Early Termination [32](#__RefHeading___Toc274753312)

4.3 Discontinuation [32](#__RefHeading___Toc274753313)

4.3.1 Subject Discontinuation [32](#__RefHeading___Toc274753314)

4.3.2 Study Discontinuation [33](#__RefHeading___Toc274753315)

5.0 ASSESSMENT OF SAFETY [34](#__RefHeading___Toc274753316)

5.1 Safety Plan [34](#__RefHeading___Toc274753317)

5.2 Adverse Events [34](#__RefHeading___Toc274753318)

5.3 Laboratory Tests/Other Evaluations [35](#__RefHeading___Toc274753319)

5.4 Procedures for Eliciting, Recording, and Reporting Adverse Events [36](#__RefHeading___Toc274753320)

5.4.1 Recording Adverse Events [36](#__RefHeading___Toc274753321)

5.4.2 Adverse Events Requiring Expedited Reporting [37](#__RefHeading___Toc274753322)

5.4.3 Special Reporting Situations [37](#__RefHeading___Toc274753323)

5.4.3.1 Death [37](#__RefHeading___Toc274753324)

5.4.3.2 Hospitalizations for Surgical or Diagnostic Procedures [37](#__RefHeading___Toc274753325)

5.5 Type and Duration of Follow-up after Adverse Events [37](#__RefHeading___Toc274753326)

5.5.1 Post Treatment Follow-up [37](#__RefHeading___Toc274753327)

5.5.2 Post Adverse Event Follow-up [38](#__RefHeading___Toc274753328)

6.0 Data Analysis and Statistical Methods [39](#__RefHeading___Toc274753329)

6.1 Disposition of the Study Subjects and Conduct of the Study [39](#__RefHeading___Toc274753330)

6.2 Determination of Sample Size [39](#__RefHeading___Toc274753331)

6.3 Analysis of Treatment Group Comparability [39](#__RefHeading___Toc274753332)

6.4 Biological and Clinical Activity Analysis [39](#__RefHeading___Toc274753333)

6.5 Safety Analysis [39](#__RefHeading___Toc274753334)

6.5.1 Adverse Events [40](#__RefHeading___Toc274753335)

6.5.2 Clinical Laboratory Evaluation [40](#__RefHeading___Toc274753336)

6.5.3 Vital Signs [40](#__RefHeading___Toc274753337)

6.6 Missing Data [41](#__RefHeading___Toc274753338)

6.7 Interim Analyses [41](#__RefHeading___Toc274753339)

6.8 Publication Policy [41](#__RefHeading___Toc274753340)

7.0 quality control and quality assurance [42](#__RefHeading___Toc274753341)

7.1 Ethical Considerations – Overall Risk and Benefit [42](#__RefHeading___Toc274753342)

7.2 Informed Consent [42](#__RefHeading___Toc274753343)

7.3 Study Monitoring Requirements [42](#__RefHeading___Toc274753344)

7.4 Disclosure of Data [42](#__RefHeading___Toc274753345)

7.5 Retention of Records [43](#__RefHeading___Toc274753346)

8.0 REFERENCES [44](#__RefHeading___Toc274753347)

# LIST OF TABLES

Table 1 Study design [20](#__RefHeading___Toc274643361)

Table 2 Composition of Drug Product (100mg Formulation) [21](#__RefHeading___Toc274643362)

Table 3 Bloodanalysis [25](#__RefHeading___Toc274643363)

Table 4 Subject Compensation [26](#__RefHeading___Toc274643364)

Table 5 Exercise Bout Blood Sample Timepoints and Tasks [29](#__RefHeading___Toc274643365)

# list of Figures

Figure 1 Anakinra/Placebo Sample Study Drug Vial Label [22](#__RefHeading___Toc274643075)

# LIST OF APPENDICES

Appendix 1 Standard Operating Procedure for the Treadmill Ergometer Test [46](#__RefHeading___Toc153007046)

Appendix 2a - Research Chart [47](#__RefHeading___Toc153007047)

Appendix 2b – Research Chart Footnotes [48](#__RefHeading___Toc153007048)

Appendix 3 Standard Operating Procedure for the 1-hour Treadmill Ergometer Exercise [49](#__RefHeading___Toc153007049)

Appendix 4 Adverse event definition and reporting guidelines [50](#__RefHeading___Toc153007050)

Appendix 5 Activity Induced Fatigue (ACTIF) Scale before and after exercise 51

Appendix 6 Beck-depression-inventory Fast Screen (BDI-FS)……………………………...53

Appendix 7 Symbol Digit Modalities Test (SDMT)…………………………………………54

Appendix 8 VAS Fatigue after Exercise……………………………………………………..56

Appendix 9 Hand-Grip-Strength-Test………………………………………………………..57

Appendix 10 Retinal microcirculation and the arteriolar to venular ratio (AVR)…………….62

Appendix 11 Cardio-ankle vascular index (CAVI) and Pulse wave velocity (PWV)………...63

Appendix 12 Activity questionnaire (Aktivitätsfragebogen)………………………………….64

LIST OF ABBREVIATIONS

| ACTIF Scale | Activity Induced Fatigue Scale |
| --- | --- |
| ADCC | Antibody dependent cell-mediated cytotoxicity |
| AE | Adverse event |
| ALT | Alanine aminotransferase |
| ANC | Absolute neutrophil count |
| ANCOVA | Analysis of covariance |
| AST | Aspartate aminotransferase |
| ATC | Acute toxic class (of WHODRUG dictionary) |
| AUC | Area under the curve |
| AVR | Arteriolar-to-venular-ratio |
| BDI-FS | Beck-Depression-Inventory Fast Screen |
| BMI | Body mass index |
| BP | Blood pressure |
| BUN | Blood urea nitrogen |
| CAVI | Cardio-ankle vascular index |
| CBC | Complete blood count |
| CHO | Chinese hamster ovary |
| CRF | Case Report Form |
| CTCAE | Common Terminology Criteria for Adverse Events |
| EC | Ethics Committee |
| ECG | Electrocardiogram |
| ECL | Electrochemiluminescence |
| GCP | Good Clinical Practice |
| GIP | Gastric inhibitory polypeptide |
| GLP | Good Laboratory Practice |
| GLP-1 | Glucagon-like peptide-1 |
| HbA1c | Test measuring the amount of glycosylated hemoglobin in blood |
| HDL | High-density lipoprotein |
| hs-CRP | High sensitivity C-reactive protein |
| ICH | International Conference on Harmonization |
| IL-1β | Interleukin-1β |
| IV | Intravenous |
| LDL | Low-density lipoprotein |
| mAb | Monoclonal antibody |
| MedDRA | Medical Dictionary for Regulatory Activities |
| MHC | Major histocompatibility complex |
| NCI | National Cancer Institute |
| NOAEL | No-observed-adverse-effect-level |
| NSAID | Non-steroidal anti-inflammatory drug |
| PD | Pharmacodynamic |
| PK | Pharmacokinetic |
| PWV | Pulse wave velocity |
| rHu | Recombinant human |
| rMu | Recombinant mouse |
| SAE | Serious adverse event |
| SC | Subcutaneous |
| SDMT | Symbol Digit Modalities Test |
| SNF | Swiss National Foundation |
| siRNA | Small interfering RNA |
| TLR | Toll-like receptor |
| T1D | Type 1 diabetes |
| T2D | Type 2 diabetes |
| USP | United States Pharmacopeia |
| ULN | Upper limit of normal |
| VAS | Visual analogue scale |
| WBC | White blood cell (count) |
| WHO DRUG | World Health Organization Drug Dictionary |

# 1.0 background

## 1.1 Overview

The role of IL-6 in regulating glucose homeostasis is unclear and the ongoing debate is characterized by IL-6 being a “bad or a good guy”. In support of the bad guy hypothesis systemic IL-6 levels are elevated in obese individuals and in patients with type 2 diabetes, and these elevated IL-6 levels predict disease development (1; 2). In addition, IL-6 induces insulin resistance in liver and fat tissues (3). The discovery that skeletal muscle produces and releases IL-6 in response to muscle contraction (exercise) has challenged the bad guy hypothesis, in addition, the fact that IL-6 improves muscle insulin sensitivity (3) supports a potential beneficial role of IL-6 in regulating glucose homeostasis. We have recently observed that IL-6 induces GLP-1 production and release from intestinal L-cells and islet α-cells (Ellingsgaard et al., manuscript in preparation), indicating that IL-6 may play an important beneficial role in regulating glucose homeostasis.

It has been shown recently that IL-1 antagonism improves glycemia and insulin secretion in patients with type 2 diabetes (4). However, IL-1 antagonism also decreases IL-6 levels in auto-inflammatory diseases including type 2 diabetes. Subsequently, IL-1 antagonism could possibly decrease GLP-1 levels and thereby reduce the beneficial effect of IL-1 antagonism on glucose-metabolism.

Exercise improves β-cell function but the underlying mechanism is unclear. Since muscle contraction leads to a strong increase in circulating IL-6 (5), we hypothesize that exercise-induced IL-6 promotes GLP-1 secretion from intestinal L-cells and islet α-cells, thereby providing a mechanism how physical activity (exercise) can help maintain and improve β-cell function in obese individuals and patients with type 2 diabetes.

However, little is known about the regulation of muscle derived IL-6 during exercise and in particular whether it is regulated by IL-1 or independently of the IL-1 system. The presence of high levels of IL-1α in the muscle makes it a candidate for IL-6 regulation.

[http://www.informaworld.com/smpp/title~db=all~content=t713926139~tab=issueslist~branches=18 - v18](http://www.informaworld.com/smpp/title~db=all~content=t713926139~tab=issueslist~branches=18#v18)Evidence is growing that an increase in inflammatory markers is directly linked with fatigue (6). IL-6 is crossing the blood-brain barrier and has been described to be associated with increased fatigue after training overload (7). Hypothesizing that IL-1 antagonism will decrease IL-6 we intend to investigate the effect of IL-1 antagonism on fatigue after exercise.

Pedersen et al. showed that skeletal muscle produces IL-6 in response to physical activity (5). IL-6 has been demonstrated to be directly involved in stress-induced hypertension (8) as well as angiotensin-II mediated hypertension (9). Hypothesizing that IL-1 antagonism will decrease IL-6 we intend to investigate the effect of IL-1 antagonism on vascular function after exercise.

## 1.2 Disease Pathophysiology

We will be evaluating the normal physiology of skeletal muscle in an exercise setting.

## 1.3 Clinical Data

A recently completed 13-week clinical study of anakinra therapy in type 2 diabetes (T2D) was based on the rationale that in vitro glucotoxicity to human β-cells can be prevented with IL-1Ra, and that glucose induces islet IL-1 production, which causes β-cell apoptosis and impaired function by pathways partly similar to those suspected to operate in T1D diabetes (4). This study provided proof-of-principle that inhibition of IL-1 signaling can improve glycemia and β-cell function. Interestingly, the maximal effect on glycemia with anakinra was seen after 4 weeks and fasting blood glucose already was significantly reduced after 1 week, suggesting rapid effects on β‑cell secretory capacity rather than changes in β‑cell mass. Long-term treatment may uncover additional beneficial effects on β‑cell mass.

## 1.4 Anakinra/Kineret®

Kineret (anakinra; r-metHuIL-1ra, Amgen Inc.) is a recombinant, nonglycoslyated form of the human interleukin-1 receptor antagonist (IL-1Ra) in a 100 mg/ml solution for s.c. injection. Kineret differs from native human IL-1Ra in that it has the addition of a single

methionine residue at its amino terminus. Kineret consists of 153 amino acids and has a molecular weight of 17.3 kilodaltons. Kineret is produced by recombinant DNA technology using an E. coli bacterial expression system.

Mild and transient injection reactions occurring in 20-50% of subjects may pose a problem in maintaining the blinding of the study, but is a similar problem in many trials using drugs with easily recognizable side-effects e.g. cyclosporine, PPARγ agonists and GLP-1. There is no alternative than to strive for the best masking possible since it is not ethically defendable to add drugs, e.g. histamine to placebo to reproduce the local adverse effects of anti-IL-1. Since only one fifth to one half of patients develops local reactions to anakinra, patients NOT developing those reactions cannot exclude that they receive active drug.

Anakinra is FDA approved for the indication rheumatoid arthritis (10,11) and has an acceptable risk / benefit profile in this indication, with more than 100.000 patients treated. Most common adverse events include mild and transient local injection reactions in 20-50% of subjects treated with Anakinra. Consistent with its mechanism of action, anakinra reduces WBC/ANC in 2.4% of patients and this may increase the risk of infection. Accordingly, treatment with anakinra will not be initiated in patients with active infections. Safety will be monitored by physical exams and blood and urine tests. Additional information is provided in the Investigator’s Brochure.

## 1.5 Study Rationale

The aim of the study is to investigate whether exercise induced increases in IL-6 are dependent on the IL-1 system. In addition, we will assess the effect of IL-1 antagonism on insulin and GLP-1 secretion as well as muscle soreness,fatigue and vascular function in response to an acute exercise bout.

Since IL-1 antagonism improves glycemia and insulin secretion in patients with type 2 diabetes it is important to investigate if in parallel to this beneficial effect it has an impact on muscle derived IL-6 with subsequent decrease in GLP-1. This may require additional medication like IL-6 substitution or DPP-IV antagonists.

# 2.0 OBJECTIVES

The primary objective of this study is to assess the regulation of muscle derived IL-6 during exercise and in particular whether it is regulated by the IL-1 system.

# 3.0 Study Design

## 3.1 Description of the Study

This is a randomized placebo-controlled, double blind, cross-over, proof-of-concept study of the effects of IL-1 antagonism on peripheral muscle physiology.

The study will consist of one screening visit followed by 3 study visits. During the first two study visits, the subjects (20 apparently healthy, lean men) will perform a submaximal exercise bout on a treadmill for 60 minutes. In addition, subjects will fill in an Activity Induced Fatigue (ACTIF) Scale before (ACTIF pre) and 3-4 hours after (ACTIF post) exercise. After every ACTIF Scale evaluation the subjects perform a Hand-Grip-Strength-Test, assessing the actual motoric shape. Then the subjects are tested by a neuropsychologist by applying the Symbol Digit Modalities test (SDMT), a cognitive screening-test to assess information processing speed and working memory. To assess depression, subjects will be examed by the Beck-Depression-Inventory Fast Screen (BDI-FS), a short version of the Beck-Depression-Inventory (BDI), depicting different facets of depression. These additional tests are performed to evaluate the impact of fatigue on cognitive, motoric and emotional behavior. Before the Activity Induced Fatigue (ACTIF) Scale after exercise (ACTIF post) is applied, subjects will be asked to evaluate their general, motoric and cognitive condition using the Visual analogue scale (VAS) Fatigue after exercise. Subjects will be randomly assigned into two groups consisting each of 10 subjects receiving study medication in a double-blinded, crossed over manner. Treatment consists of a single subcutaneous injection of 100mg IL-1Ra or placebo at visit 1 and 2.,

[visit 1: IL-1Ra/ placebo; visit 2: placebo/ IL-1Ra (cross-over to day 1);]. See table 1 on page 18.

Additionally the vascular function will be evaluated before the injection of the study medication and 1 hour after the exercise bout. The large arterial stiffness known as cardio ankle vascular index (CAVI) and the pulse wave velocity (PWV) will be measured by an oscillometric sphygmograph (Vasera). Furthermore the small vessels will be examined by taking a picture from the retina to calculate the arteriolar-to-venular-ratio (AVR). To assess overall activity of the participants will be given an activity questionnaire (Aktivitätsfragebogen) at screening (12).

The screening visit and the three study visits will be separated by at least 72 hours and at most 9 days.

.

## 3.2 Rationale for Study Design and Dose Level

### 3.2.1 Rationale for Dose Level

#### **3.2.1.1 Anakinra/Kineret®**

The standard dose of anakinra is 100 mg, without adjustment for body weight. For a more complete discussion, please refer to the current Investigator’s Brochure.

### 3.2.2 Rationale for Study Design

The 20 subjects in this study will receive SC doses of 100 mg anakinra or placebo. The size of the active and placebo groups are considered adequate for an exploratory appraisal of the effects of treatment with anakinra on peripheral muscle physiology.

## 3.3 Selection of Study Population

### 3.3.1 Subject Recruitment

Subjects will be recruited from the local population by internet ([www.endo-diabasel.ch](http://www.endo-diabasel.ch/), [www.markt.unibas.ch](http://www.markt.unibas.ch/)) and newspaper advertisement (Baslerstab, 20Minuten). Subjects responding to the advertisement will be screened by a telephone interview. Those deemed eligible will be invited for a screening visit.

### 3.3.2 Inclusion Criteria

Subjects may be included in the study if they meet all of the following criteria:

- male
- non-smoking
- apparently healthy
- BMI >18 and <26kg/m2
- Age 20-50 years
- Regular exercise including a minimum of two runs weekly of a total duration of > 2h
- Willingness to use contraceptive measures adequate to prevent the subject’s partner from becoming pregnant during the study. Adequate contraceptive measures include hormonal methods used for two or more cycles prior to Screening (e.g., oral contraceptive pills, contraceptive patch, or contraceptive vaginal ring), double barrier methods (e.g., contraceptive sponge, diaphragm used in conjunction with contraceptive foam or jelly, and condom used in conjunction with contraceptive foam or jelly), intrauterine methods (IUD), sterilization (e.g., tubal ligation or a monogamous relationship with a vasectomized partner), and abstinence.

### 3.3.3 Exclusion Criteria

Subjects will be excluded from the study if they meet any of the following criteria:

- Clinical signs of infection in the week before inclusion or history of infection during the last 3 months (CRP >5mg/L)
- Impaired fasting glucose (fasting plasma glucose >5.5mmol/L)
- Hematologic disease (leukocyte count < 1.5x10^9^/L, hemoglobin <11 g/dL, platelets <100 x 10^3^/uL)
- Kidney disease (creatinine > 1.5 mg/dL for men and 1.4mg/dL for woman)
- Liver disease (transaminases >2x upper normal range)
- Heart disease
- Pulmonary disease
- Inflammatory disease
- History of carcinoma
- History of tuberculosis
- Alcohol consumption >40g/d
- Known allergy to Kineret
- Current treatment with any drug in the week before inclusion, including vitamin supplementation (especially vitamin C and E)
- Use of any investigational drug within 30 days prior to enrollment or within 5 half-lives of the investigational drug, whichever is longer
- Subject refusing or unable to give written informed consent

## 3.4 Prior and Concomitant Therapy

Concomitant medications are prohibited during the specified time periods:

## 3.5 Enrollment, Treatment Assignment, Blinding, and Unblinding

### 3.5.1 Enrollment

Once Screening is completed and subject eligibility is confirmed, a subject will be assigned a subject number and a kit number(s) according to the randomization schedule. Enrolled subjects who discontinue the trial prior to receiving study drug will be replaced. Subjects who discontinue after receiving any amount of study drug will not be replaced (see Section 4.3.1).

### 3.5.2 Treatment Assignment

Subjects who meet the study eligibility requirements and have signed the informed consent will be randomly assigned to receive study medication according to the following chart:

Table 1
Study design

|  | **1** | **2** |
| --- | --- | --- |
| 20 Participants | Group 1: (n=10)  **IL-1Ra** | Group 1: (n=10)  **Placebo** |
|  | Group 2: (n=10)  **Placebo** | Group 2: (n=10)  **IL-1Ra** |

### 3.5.3 Treatment Blinding

This is a blinded study in which the subject, as well as the principal investigator, study coordinator, and other site personnel will have no knowledge of subject treatment assignments for the duration of the trial. The study is being blinded in this way to prevent bias in the safety evaluation, in particular the severity grading and causality determinations for AEs. The pharmacist may become unblinded during visual inspection of study drug. After all subjects have completed Final Visit assessments, the data will be cleaned and locked, and personnel will be unblinded in order to complete analysis of data. The clinical Trial Unit (CTU) of the University Hospital Basel, Switzerland, will be responsible for treatment blinding and preparation of trial drugs throughout the study.

All active and placebo vials will be randomized with a kit number. Subjects will be assigned a unique subject number and kit number (or kit numbers if they require multiple vials) according to the randomization schedule. The label on the vial will indicate the study number and kit number but will not indicate the treatment assignment. Subject number will be filled in on the label upon randomization.

After preparation the color, consistency and amount of Verum/Placebo (on visits 1 and 2) will be indistinguishable.

The randomization schedule will be kept by an independent outside biostatistician, as well as the company responsible for packaging, labeling, distribution of the vials, and assignment of kits to subjects.

The investigator must contact the CTU if it is necessary to break the blind (see Section 3.5.4).

### 3.5.4 Treatment Unblinding

Unblinding of individual treatment assignment will occur when medically necessary.

## 3.6 Treatment

### 3.6.1 Formulation of Anakinra/Kineret®

Anakinra will be commercially acquired. The matching placebo for Anakinra will be saline (NaCl 0.9%).

The composition of the Anakinra drug product formulation is provided in Table 2

Table 2
Composition of Drug Product
(100mg Formulation)

| Ingredient | Concentration |
| --- | --- |
| r-metHuIL-1ra (Anakinra) | 150mg/mL |
| Natriumcitrat |  |
| Natriumchlorid |  |
| Natriumedetat |  |
| Polysorbat 80 |  |
| Natriumhydroxid |  |
| Wasser für Injektionszwecke |  |

For further details on the formulation of Anakinra/Kineret®, see the Investigator’s Brochure.

### 3.6.2 Labeling

All vial labels will include the information specified in Figure 1 (on visits 1 and 2) .

Figure 1
Anakinra/Placebo Sample Study Drug Vial Label

| *Protokoll-Nr.:* 9.0 **Anakinra oder Placebo für Anakinra**  Inhalt: 1 × 1.0 ml | | | | | | |
| --- | --- | --- | --- | --- | --- | --- |
|  | Steriles Lösung für subkutanen Gebrauch Dosierung und Verabreichung: 1.0ml s.c. | | | | |  |
| Probanden-ID:  _________ | | | Chargen-Nr.: JXXXX | *Zubereitet durch / Datum:*  ______________ | | |
| Überprüfungstermin: xx/xx Lagerung bei: 2 – 8°C (36 – 46°F) | | | | | | |
| VORSicht Nur zur Verwendung in der klinischen Studie | | | | | | |
|  | | **Sponsor:**  Prof. M. Donath, UniversitätsSpital Basel, Petersgraben 4, CH-4031 Basel, Schweiz, +41 (0) 61 2652525 | | |  | |

### 3.6.3 Shipping of study drugs

Anakinra will be purchased locally at the Spitalpharmazie of the University Hospital Basel

### 3.6.4 Dosage, Preparation, Administration, and Storage

#### 3.6.4.1 Dosage

Each subject will receive one SC injections of study drug (100 mg anakinra or matching placebo) on visits 1, 2

#### 3.6.4.2 Preparation

Study drug will be prepared by a pharmacist or designated medical professional prior to administration.

#### 3.6.4.3 Administration

SC injections will be administered by a trained health care professional. Injections should be given in the anterior abdomen. The injection site will first be cleaned with an alcohol swab, and the alcohol will be allowed to dry prior to the injection.

On all study drug administration visits, all vital signs other than height and weight are recorded within 15 minutes prior to the injection, every 30 ± 10 minutes following the injection until 3 hours after the injection, and immediately prior to the subject’s release. Subjects will not be discharged from the facility until at least 2 hours after the injection and at least 1 hour after vital signs are stable. If a subject experiences a serious adverse event (SAE), vital signs may be taken more frequently, per standard institutional practice. In the period following the injection, the responsible physician will be notified immediately should any of the following conditions apply:

- Systolic blood pressure is persistently (after 3 measurements) more than 20 mm Hg above or below baseline (dosing day, pre‑dose).
- Pulse is more than 20 beats per minute above or below baseline (dosing day, pre‑dose) or if pulse is irregular.
- Temperature is > 38.3°C.
- Signs of anaphylaxis, including respiratory distress (dyspnea, oxygen desaturation with PO_2_ < 90% or onset of acute respiratory distress syndrome), flank or back pain, and/or hypotension. If any of these symptoms occur, appropriate therapy (e.g., oxygen, epinephrine, diphenhydramine, and corticosteroids) will be administered. Complete documentation of the event will be recorded.

#### 3.6.4.4 Storage and Handling

Study drug will be stored refrigerated at 2–8°C (36–46°F) and protected from direct sunlight until time of use. Aseptic techniques will be used during withdrawal, preparation, and administration.

Any study drug remaining in the vial once the subject’s dose has been prepared will not be discarded but will be stored in a secured area until a drug accountability has been performed.

#### 3.6.4.5 Drug Accountability

Anakinra/Kineret® and Placebo required for this study will be acquired commercially as mentioned in section 4.0. The site must maintain accurate records of all study drug received, dispensed, returned, or destroyed. Upon completion of the study, all study drug must be accounted for.

## 3.7 Selection of Doses Used in the Study

Subjects who receive active treatment will receive SC injections of 100 mg of anakinra . The rationale for the selected dose regimen is provided in Section 3.2.1.

## 3.8 Outcome Measures

### 3.8.1 Primary Outcome Measure

The primary endpoint is change in exercise-stimulated IL-6 between group dosed with placebo and anakinra.

### 3.8.2 Secondary Outcome Measures

The secondary endpoints are as follows:

- Change in glucose metabolism, insulin glucagon and GLP-1 secretion due to any treatment.
- Change of inflammatory markers (CRP, TNFα, IL-1Ra) due to any treatment.
- Change of stress hormones (cortisol, growth hormone, catecholamines) due to any treatment.
- Change of muscular enzymes (CK) due to any treatment.
- Well-being during and after exercise including muscle soreness due to any treatment.
- Activity Induced Fatigue (ACTIF) Scale before and after exercise due to any treatment.
- Depression
- Cognition
- Motor strength
- Change of vascular function (CAVI, PWV and AVR) due to any treatment.

The tests used to investigate the regulation of IL-6 during exercise are summarized in Table 3.

Table 3
Bloodanalysis

Blood samples will be drawn at the following time points: -60 min before, 0, 10, 20, 30, 40, 50, 60 min during exercise and at +10, +20, +30 and + 60 min after exercise.

A total of 250 ml blood will be drawn throughout the study.

The following parameters will be assessed:

| Time points: | -60 | 0 | 10 | 20 | 30 | 40 | 50 | 60 | +10 | +20 | +30 | +60 |
| --- | --- | --- | --- | --- | --- | --- | --- | --- | --- | --- | --- | --- |
|  | **before** | **exercise** | | | | | | | **after** | | | |
| IL-6 | x | x | x | x | X | x | x | x | x | x | x | x |
| GLP-1 (active) | x | x | x | x | X | x | x | x | x | x | x | x |
| Glucagon | x | x | x | x | X | x | x | x | x | x | x | x |
| Insulin | x | x | x | x | X | x | x | x | x | x | x | x |
| Glucose | x | x | x | x | X | x | x | x | x | x | x | x |
| IL-1Ra | x |  |  | x |  | x |  | x | x |  | x |  |
| GIP | x |  |  | x |  | x |  | x | x |  | x |  |
| usCRP | x |  |  | x |  | x |  | x | x |  | x |  |
| TNF-a | x |  |  | x |  | x |  | x | x |  | x |  |
| Cortisol | x |  |  | x |  | x |  | x | x |  | x |  |
| GH | x |  |  | x |  | x |  | x | x |  | x |  |
| CK | x |  |  |  |  |  |  | x |  |  |  | x |

### 3.8.3 Safety Outcome Measures

Safety will be assessed by the following outcome measures:

- AEs
- Clinically relevant abnormal changes in vital signs compared to baseline (Day 0 pre‑dose)
- Clinically relevant abnormal changes in laboratory values (hematology, chemistry, and urinalysis) compared to baseline (Day 0 pre‑dose)
- Dosing reactions (e.g., fevers, chills, relevant changes in vital signs, or hives within 4 hours of dosing)

## 3.9 Subject costs and insurance

All assessments mentioned in this protocol as well as the study drugs are free of charge for the subject. This study will not cause personal or insurance related costs. In case of damage all research subjects are covered by the University Hospital Basel.

## 3.10 Subject Compensation

All subjects will be given financial compensation according to table 4

Table 4
Subject Compensation

| ***Visit*** | *0 (screen)* | *1* | *2* | *3* |
| --- | --- | --- | --- | --- |
| **Payment in SFR.** | 20 | 90 | 90 | 30 |

## 3.11 Financing

The study will be financed by the SNF.

# 4.0 STUDY OPERATIONS and Evaluations

Dr. Marc Donath at the University Hospital Basel both the principal investigator and sponsor of this trial. Dr. Marc Donath is supplying Anakinra/Kineret®, which is commercially available.

## 4.1 Reporting and Recording of Data

The CRFs will be completed according International Conference on Harmonization/Good Clinical Practice (ICH/GCP) Guidelines.

A CRF will be completed for each subject enrolled under this protocol. Information collected on the subjects who fail screening will include date of birth, and the reason the subject was not enrolled.

All information collected in the CRF will be verifiable in the source documents. Source documents for this trial will include any study-specific worksheets or laboratory result reporting documents. Subject medical records will contain reference to the study title and assigned subject identification number. The signed consent form will be filed with the subject’s medical record.

CRF completion will be kept current to reflect subject status during the course of the trial. All CRF data will be reviewed, signed, and dated by the principal investigator.

Study subjects will be identified by subject ID number and date of birth.

## 4.2 Study Assessments

Study assessments are shown in the Schedule of Assessments (Appendix 2). Routine laboratory analyses, including chemistries, complete blood count (CBC), coagulation measures, urinalysis, and diabetes-specific analyses will be performed locally.

### 4.2.1 Screening Assessments (Day -28 to -7)

Subjects potentially meeting the inclusion criteria as determined over a pre-screening telephone call will be scheduled for their screening visit. The screening will take place 28 to 7 days prior to the first study procedure day. For the screening visit the selected subject will be given an appointment and instructed to come in after an overnight fast of 6-10 hours. If the subject fails to fast 6 – 10 hours prior to visit, appointment will be rescheduled to the next possible date. The following procedures and assessments will be conducted:

- Study investigators will obtain written informed consent before blood sampling from eligible subjects at the beginning of this same visit.
- Demographics (including age, sex and race)
- Medical history
- Concomitant medications
- Vital signs (heart rate, blood pressure (systolic/diastolic, measured 3 times), respiratory rate, temperature (oral or tympanic), s0_2_, height, weight, BMI, medical history and a complete physical examination will be performed.
- Electrocardiogram (ECG)
- Complete blood count (CBC) with differential, platelets
- Serum chemistry including sodium, potassium, creatinine, CRP, liver transaminases, fasting plasma glucose, AP, CK
- Spontaneous urine sample (strip for the presence of protein, leucocytes and erythrocytes)
- Registration of right thigh (15cm above patella) and right upper arm (10cm above epicondylus lateralis) diameter in order to standardize muscle mass
- For subjects with reproductive potential, a willingness to use contraceptive measures adequate to prevent the subject’s partner from becoming pregnant during the study. Adequate contraceptive measures include hormonal methods used for two or more cycles prior to Screening (e.g., oral contraceptive pills, contraceptive patch, or contraceptive vaginal ring), double barrier methods (e.g., contraceptive sponge, diaphragm used in conjunction with contraceptive foam or jelly, and condom used in conjunction with contraceptive foam or jelly), intrauterine methods (IUD), sterilization (e.g., tubal ligation or a monogamous relationship with a vasectomized partner), and abstinence.
- Inclusion/exclusion
- A treadmill ergometer test will determine individual heart rate (HR)-VO2 relationships, and VO2max on which the exercise load for the acute exercise bout will be based (for further information see Appendix 1)

### 4.2.2 Assessments for Treatment and Follow-up Period

#### 4.2.2.1 Visit 1 (Day 0)

Subjects must fast 6 to 10 hours prior to the visit. If the subject fails to fast 6 – 10 hours prior to visit, appointment will be rescheduled to the next possible date. The subjects will be asked to fill in the Activity Induced Fatigue (ACTIF) Scale ACTIF pre. Afterwards the subjects perform the Hand-Grip-Strength-Test, the Symbol Digit Modalities Test (SDMT) and the Beck-Depression-Inventory Fast Screen (BDI-FS). Then the vascular function will be analysed by Vasera and SVA. An intravenous catheter for blood drawings will be placed in the forearm/elbow, 5 minutes prior to first time point for blood drawing. 60 minutes before exercise, the first blood sample will be drawn. Right after the first blood sample has been taken, subjects will receive a single subcutaneous injection of **100mg anakinra or placebo**. Directly after the injection the vascular function will again be analysed by Vasera and SVA. Between blood draws, the IV catheters will be kept patent by a slow infusion of saline. Prior to starting the 1-hour exercise the subject is informed about the procedure. A heart rate monitor is placed around the chest and, at time 0, the subject begins to run, starting with a 5 min warm up period at 2 to 4 km/h under target speed at an incline of 0.5%. After 5 min, and in continuation of warm up, treadmill speed is increased so the subject reaches 75% of VO_2max_ based on heart rate measurements, and this submaximal workload is maintained for the remaining time (approx. 55 min, total running time 60 min).

For blood sampling, we will lower the speed of the treadmill to walking speed in order to be able to draw the blood without any complications. The total exercise time per session will be 60 minutes. After finishing the exercise, the subject will stay on the treadmill for a “cool down” at walking speed for 5 min. For the remaining time, until finishing with the blood drawing, subjects will be seated on a chair. Directly after the exercise and after the last blood drawing (1 hour after finishing exercise) the evaluation of the vascular function by Vasera and SVA will be repeated. After the last time point for blood drawing (60 minutes after exercise) the intravenous catheter will be removed. Since subjects have been fasting prior to the exercise bout, we will provide them with a sandwich and a cold drink after finishing with the last blood drawing. If, 15minutes after the last blood drawing, subjects show no signs of malaise they will be given information concerning their next exercise bout and are free to go. 3-4 hours after finishing exercise subjects will return to the study facility. The subjects will be asked to assess their actual overall, motoric and cognitive condition using the VAS fatigue after exercise visual analogue scale. Afterwards they are asked to fill in the Activity Induced Fatigue (ACTIF) Scale ACTIF post. Then the subjects perform the Hand-Grip-Strength-Test, the Symbol Digit Modalities Test (SDMT) and the Beck-Depression-Inventory Fast Screen (BDI-FS) test. Thereafter the subjects are free to go.

Table 5
Exercise Bout Blood Sample Timepoints and Tasks

| **Timepoints** | **Tasks** |
| --- | --- |
| -65min (before exercise) | Intravenous catheter for blood drawing |
| -60 | Blood Sample Nr. 1, then s.c. study drug injection |
| 0 (start of exercise) | Blood Sample Nr.2 |
| 10 | Blood Sample Nr.3 |
| 20 | Blood Sample Nr.4 |
| 30 | Blood Sample Nr.5 |
| 40 | Blood Sample Nr.6 |
| 50 | Blood Sample Nr.7 |
| 60 | Blood Sample Nr.8 |
| +10 (after exercise) | Blood Sample Nr.9 |
| +20 | Blood Sample Nr.10 |
| +30 | Blood Sample Nr.11 |
| +60 | Blood Sample Nr.12, then removal of i.v. catheter |
| +75 | Subjects are free to go if they show no signs of malaise |

After all procedures for this visit have been completed, subjects should resume medications as previously prescribed.

All exercise will be carried out with a heart rate monitor in order to document and control exercise intensity.

Blood samples will be centrifuged and the plasma stored at -80°C prior to being assayed.

The following procedures and assessments will be conducted pre-dose unless otherwise indicated:

- Interim medical history
- Concomitant medications
- Vital signs including pulse, blood pressure (systolic/diastolic), respiratory rate, temperature (oral or tympanic), p0_2_, and body weight. Weight is measured pre-dose to calculate the study drug dose to be administered throughout the study. All vital signs other than weight are recorded within 15 minutes prior to the injection, every 30 ± 10 minutes following the injection until 2 hours after the injection, and immediately prior to the subject’s release. Subjects will not be discharged from the facility on Day 0 until at least 2 hours after the injection and at least 1 hour after vital signs are stable. If a subject experiences an SAE, vital signs may be taken more frequently, per standard institutional practice.
- Activity Induced Fatigue Scale ACTIF pre
- SDMT
- BDI-FS
- Hand-Grip-Strenth-Test
- Retrospective analysis blood sample
- First blood sample
- Study drug administration
- Exercise bout (post-dose)
- AEs (post-dose)

#### 4.2.2.2 Visit 2 [Day 6 (± 3 Days)]

Subjects must fast 6 to 10 hours prior to the visit. If the subject fails to fast 6 – 10 hours prior to visit, appointment will be rescheduled to the next possible date. The subjects will be asked to fill in the Activity Induced Fatigue (ACTIF) Scale ACTIF pre. Afterwards the subjects perform the Hand-Grip-Strength-Test, the Symbol Digit Modalities Test (SDMT) and the Beck-Depression-Inventory Fast Screen (BDI-FS) test. Then the vascular function will be analysed by Vasera and SVA. An intravenous catheter for blood drawings will be placed in the forearm/elbow, 5 minutes prior to first time point for blood drawing. 60 minutes before exercise, the first blood sample will be drawn. Right after the first blood sample has been taken, subjects will receive a single subcutaneous injection of **100mg anakinra or placebo** (crossover from Visit 1). Between blood draws, the IV catheters will be kept patent by a slow infusion of saline. Prior to starting the 1-hour exercise the subject is informed about the procedure. A heart rate monitor is placed around the chest and, at time 0, the subject begins to run starting with a 5 min warm up period at 2 to 4 km/h under target speed at an incline of 0.5%. After 5 min, and in continuation of warm up, treadmill speed is increased so the subject reaches 75% of VO_2max_ based on heart rate measurements, and this submaximal workload is maintained for the remaining time (approx. 55 min, total running time 60 min). For blood sampling, we will lower the speed of the treadmill to walking speed in order to be able to draw the blood without any complications. The total exercise time per session will be 60 minutes. After finishing the exercise, the subject will stay on the treadmill for a “cool down” at walking speed for 5 min. For the remaining time, until finishing with the blood drawing, subjects will be seated on a chair. Directly after the exercise and after the last blood drawing (1 hour after finishing exercise) the evaluation of the vascular function by Vasera and SVA will be repeated.After the last time point for blood drawing (60 minutes after exercise) the intravenous catheter will be removed. Since subjects have been fasting prior to the exercise bout, we will provide them with a sandwich and a cold drink after finishing with the last blood drawing. If, 15minutes after the last blood drawing, subjects show no signs of malaise they will be given information concerning their next exercise bout and are free to go. 3-4 hours after finishing exercise subjects will return to the study facility. The subjects will be asked to assess their actual overall, motoric and cognitive condition using the VAS fatigue after exercise visual analogue scale. Afterwards they are asked to fill in the Activity Induced Fatigue (ACTIF) Scale ACTIF post. Then the subjects perform the Hand-Grip-Strength-Test, the Symbol Digit Modalities Test (SDMT) and the Beck-Depression-Inventory Fast Screen (BDI-FS). Thereafter the subjects are free to go.

After all procedures for this visit have been completed, subjects should resume medications as previously prescribed.

All exercise will be carried out with a heart rate monitor in order to document and control exercise intensity.

Blood samples will be centrifuged and the plasma stored at -80°C prior to being assayed.

The following procedures and assessments will be conducted pre-dose, unless otherwise indicated:

- AEs (pre-and post‑dose)
- Concomitant medications
- Muscle soreness
- Vital signs including pulse, blood pressure (systolic/diastolic), respiratory rate, temperature (oral or tympanic), p0_2_, and body weight. Weight is measured pre-dose. All vital signs other than weight are recorded within 15 minutes prior to the injection, every 30 ± 10 minutes following the injection until 1 hour after the injection, and immediately prior to the subject’s release. Subjects will not be discharged from the facility until at least 3 hour after the injection. If a subject experiences an SAE, vital signs may be taken more frequently, per standard institutional practice.
- Activity Induced Fatigue Scale ACTIF pre
- SDMT
- BDI-FS
- Hand-Grip-Strength-Test
- Retrospective analysis blood sample
- First blood sample
- Study drug administration
- Exercise bout (post-dose)
- AEs (post-dose)

#### 4.2.2.3 Visit 3 [Day 12 (±9 Days)]

Subjects must fast for 6 to 10 hours prior to the clinic visit. If the subject fails to fast 6 – 10 hours prior to visit, appointment will be rescheduled to the next possible date. After all procedures for this visit have been completed, subjects should resume medications as previously prescribed.

The following procedures and assessments will be conducted:

- AEs
- Concomitant medications
- Muscle soreness
- Vital signs including pulse, blood pressure (systolic/diastolic), p0_2_, respiratory rate, temperature (oral or tympanic), and weight (including calculation of BMI)
- Complete physical exam
- CBC with differential, platelets
- Serum chemistry including sodium, potassium, creatinine, CRP, liver transaminases, fasting plasma glucose, AP, CK
- Retrospective analysis blood sample

#### 4.2.2.4 Early Termination

Subjects who leave the study after receiving any amount of study drug should return to the clinic and complete the evaluations listed below. The reason(s) for a subject’s early termination must be clearly documented in the subject’s medical records and on the appropriate page of the CRF. Subjects must fast for at least 6-10 hours prior to the day of the clinic visit. If the subject fails to fast 6 – 10 hours prior to visit, appointment will be rescheduled to the next possible date. After all procedures for this visit have been completed, subjects should resume medications as previously prescribed.

- AEs
- Concomitant medications
- Vital signs including pulse, blood pressure (systolic/diastolic), respiratory rate, p0_2_, temperature (oral or tympanic), and weight (including calculation of BMI)
- Complete physical exam
- CBC with differential, platelets
- Serum chemistry including sodium, potassium, creatinine, CRP, liver transaminases, fasting plasma glucose, AP, CK
- Retrospective analysis blood sample if subject terminated prior to Day 12

## 4.3 Discontinuation

### 4.3.1 Subject Discontinuation

Subjects may withdraw or be withdrawn from the study at any time.

Subjects who discontinue participation in the study prior to receiving study drug will be replaced. No follow-up or additional procedures will be performed on subjects discontinuing prior to receiving study drug. Subjects who discontinue participation in the study after receiving any amount of study drug will not be replaced. However, when subject non‑compliance could adversely affect the integrity of the analyses, additional subjects may be added to the study.

If a dosed subject discontinues, every effort will be made to complete as many subsequent protocol evaluations as possible. At minimum, a subject will be followed for safety for at least 2 weeks following his final dose of study drug. If a subject discontinues and does not return to the clinic, we will attempt to maintain monthly telephone contact through Day 12. If the subject gives a reason for discontinuation, it will be clearly documented in the subject’s medical records and on the appropriate page of the CRF.

### 4.3.2 Study Discontinuation

This study may be terminated at any time. Reasons for terminating the study may include the following:

The incidence or severity of AEs indicates a potential health hazard to subjects;

Subject enrollment is unsatisfactory

# 5.0 ASSESSMENT OF SAFETY

## 5.1 Safety Plan

Following a comprehensive baseline evaluation, each subject’s safety will be monitored with periodic physical examinations, laboratory tests (hematology, chemistry, urinalysis), and the recording and evaluation of all treatment-emergent AEs.

Once evidence of a clinical and/or laboratory abnormality is noticed, the condition will be treated while trying to determine its cause. The subject will then be followed until the condition resolves or becomes chronic or stable.

Subjects will be instructed about possible acute AEs and allergic reactions, including anaphylaxis, which can occur with exogenously administered foreign antibodies. If such reactions are observed, the subjects should be promptly treated according to standard of care.

## 5.2 Adverse Events

The occurrence of an AE will be determined based on observed or volunteered signs and symptoms, as well as changes in the subject’s physical examination and laboratory results. AEs that occur during the study will be recorded on the appropriate Adverse Event pages of the CRF.

An AE is defined as any untoward medical occurrence (e.g., sign, symptom, disease, syndrome, intercurrent illness, abnormal laboratory finding) that emerges or worsens relative to baseline (Day 0 pre-dose), regardless of the suspected cause. Untoward medical events that occur from the time the subject signs the informed consent form to the time the administration of the drug starts are not considered AEs, and should be recorded under medical history.

All AEs will be evaluated for their seriousness, severity, relationship to study drug, and outcome.

An AE occurring **at any dose** (including overdose) should be classified as **SERIOUS** if:

- It resulted in death (i.e., the AE caused or led to death).
- It was life threatening (i.e., the AE placed the subject at immediate risk of death; an AE should not be classified as life-threatening if it hypothetically might have caused death if it were more severe).
- It required or prolonged inpatient hospitalization (i.e., the AE required at least a 24‑hour inpatient hospitalization or prolonged a hospitalization beyond the expected length of stay; hospitalizations for elective medical/surgical procedures, scheduled treatments, or routine check-ups are not SAEs by this criterion).
- It was disabling (i.e., the AE resulted in a substantial disruption of the subject’s ability to carry out normal life functions).
- It resulted in a congenital anomaly/birth defect (i.e., an adverse outcome in a child or fetus of a subject exposed to the molecule or study drug prior to conception or during pregnancy).
- It does not meet any of the above criteria for a serious AE but may jeopardize the subject or may require medical or surgical intervention to prevent one of the outcomes listed above.

All AEs will be graded for severity according to the National Cancer Institute Common Terminology Criteria for Adverse Events (NCI CTCAE), version 3.0 (available on line at <http://ctep.cancer.gov/reporting/ctc.html>).

The following criteria will be used to assess whether there is a reasonable possibility that the study drug caused or contributed to the AE:

Yes (possibly, probably, or definitely related):

- There is a clinically plausible time sequence between onset of the AE and study drug administration

and/or

- There is a biologically plausible mechanism for study drug causing or contributing to the AE

No (unlikely, unrelated):

- Another cause of the AE is most plausible

and/or

- A clinically plausible temporal sequence is inconsistent with the onset of the AE and study drug administration

and/or

- A causal relationship is considered biologically implausible

## 5.3 Laboratory Tests/Other Evaluations

This study involves various procedures and tests, performed repeatedly throughout the study. If a subject has a clinically significant abnormal laboratory test value that is not an expected result of study drug administration or was not present at baseline (Day 0 pre‑dose), the test will be repeated and the subject will be followed until the test value has returned to the normal range or the investigator has determined that the abnormality is chronic or stable.

All abnormal laboratory results will be evaluated for their clinical relevance. An isolated abnormal laboratory result in the absence of any associated clinical finding may or may not be considered an AE; the evaluation will be based on a consideration of the overall clinical context. An abnormal laboratory result will be considered clinically relevant and recorded as an AE when it is part of a clinical abnormality requiring specific medical intervention or follow-up.

A complete physical examination will be performed at Screening, and on Visit 3. Vital signs will be recorded at specified time points throughout the study (see Appendix 2). Vital sign measurements include pulse, blood pressure (systolic/diastolic), respiratory rate, temperature (oral or tympanic), p0_2_, weight and, at Screening only, height to enable calculation of BMI.

All samples will be stored for at least five years. The subject has the right to eliminate the samples at any time.

## 5.4 Procedures for Eliciting, Recording, and Reporting Adverse Events

AEs will be directly observed or elicited using open-ended or directed questions, and/or volunteered by the subjects.

Muscle soreness and adverse events will be assessed 24, 48 and 72 hours after every exercise bout. There will be no assessment of adverse events (AE) or muscle soreness after screening visit.

**Muscle soreness** will be assessed using a visual analog scale. This scale is standard in rheumatoid arthritis studies with slight adjustments (numbers were added in order to quantify assessment). Participants will be asked to fill out as below:


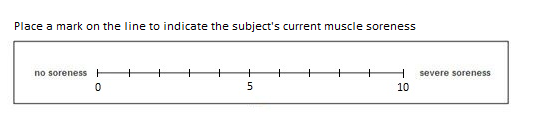


### 5.4.1 Recording Adverse Events

To improve the quality and precision of acquired AE data, the following guidelines will be used:

- Whenever possible, recognized medical terms will be used when recording AEs on the AE pages of the CRF. Colloquialisms and/or abbreviations will not be used.
- If known, the diagnosis (i.e., disease or syndrome) rather than component signs and symptoms will be recorded on AE pages of the CRF. However, signs and symptoms that are considered unrelated to an encountered syndrome or disease will be recorded as individual AEs on the CRF (e.g., if congestive heart failure has not been documented, each symptom in the above example will be recorded as a separate AE).
- AEs occurring secondary to other events (e.g., sequelae) will be identified by the primary cause. A “primary” AE, if clearly identifiable, generally represents the most accurate clinical term to record on AE pages of the CRF. Events occurring secondary to the primary event should be described in the narrative description of the case. For example:

| Orthostatic hypotension | → | Fainting and fall to floor | → | Head trauma | → | Neck pain |
| --- | --- | --- | --- | --- | --- | --- |

The primary AE in this example is orthostatic hypotension.

### 5.4.2 Adverse Events Requiring Expedited Reporting

All SAEs will be carefully monitored throughout the course of the study.

If a fatal and life-threatening SAE is evaluated as a sudden unexpected serious adverse reactions (SUSAR.) Swissmedic and KEK have to be informed without delay and no later than 7 calendar days following awareness that event meets SUSAR criteria. If a non-fatal, non-life-threatening SAE is evaluated as SUSAR then Swissmedic must be informed no later than 15 calendar days following awareness that event meets SUSAR criteria.

For further details see adverse event reporting guidelines in appendix 4.

### 5.4.3 Special Reporting Situations

#### 5.4.3.1 Death

Death is an outcome of an event. The **event** that resulted in the death will be recorded and reported on the AE page of the CRF.

#### 5.4.3.2 Hospitalizations for Surgical or Diagnostic Procedures

The illness leading to the surgical or diagnostic procedure will be recorded as the SAE, not the procedure itself. The procedure will be captured in the case narrative as part of the action taken in response to the illness.

## 5.5 Type and Duration of Follow-up after Adverse Events

### 5.5.1 Post Treatment Follow-up

All subjects receiving any amount of drug will be followed until Day 12. All protocol-defined AEs occurring during the study should be fully evaluated, recorded on the appropriate pages of the CRF, and documented in the subjects’ files.

### 5.5.2 Post Adverse Event Follow-up

All subjects who experience AEs will be followed until the event resolves or until the subject’s participation in the study ends. For those AEs judged to be unrelated to study drug, the outcome at last observation will be recorded on the appropriate pages of the CRF.

Subjects experiencing AEs that were judged to be possibly, probably, or definitely related to study drug will be followed until the events resolve or until the event to be chronic or stable. Resolution of such events will be documented on the appropriate pages of the CRF.

# 6.0 Data Analysis and Statistical Methods

Data will be analyzed for safety and biological endpoints. Safety analyses will include all subjects who received any amount of study drug. Efficacy analyses will include all randomized subjects. Analyses of the population of subjects who completed treatment may also be performed.

Since the sample size is small, only exploratory statistical testing will be performed. Study results will be summarized using primarily descriptive statistics and 95% confidence intervals. Since this is a proof of concept study, evaluating an issue that has never been examined before, it is not possible to perform a power calculation.

## 6.1 Disposition of the Study Subjects and Conduct of the Study

The disposition of subjects will be described with summaries by treatment assignment of the number of subjects enrolled, the number of subjects treated, and the number of subjects for whom study drug was permanently discontinued (including the reasons).

## 6.2 Determination of Sample Size

The sample size (20 subjects receiving treatment) was based on clinical and practical considerations.

## 6.3 Analysis of Treatment Group Comparability

Demographic and baseline characteristics will be summarized by treatment group.

## 6.4 Biological and Clinical Activity Analysis

All randomized subjects will be considered for analysis. Efficacy endpoints will be described using the five-number summary (mean, standard deviation, median, minimum, and maximum) by treatment and study visit. Graphs will also be employed.

The underlying assumptions of the t-test for the treatment group effect will be checked using the residuals from the ANCOVA model. If these assumptions are violated, the Wilcoxon signed ranks test will be used to compare treatment groups.

## 6.5 Safety Analysis

Safety analyses will involve examination of the incidence, severity, and type of treatment‑emergent AEs reported, and changes in vital signs and laboratory test results from baseline (Day 0 pre-dose) to specified time points throughout the study.

### 6.5.1 Adverse Events

Treatment-emergent AEs reported during the study will be coded using the MedDRA dictionary. An AE will be summarized by dose group and the following:

- System organ class and preferred term
- System organ class and severity
- System organ class, preferred term, and severity

These summaries will be presented for the following subsets:

- All AEs
- Drug-related AEs
- AEs resulting in discontinuation of study drug or the study
- SAEs, including deaths

Hypoglycemic events will be summarized descriptively, with summaries provided for the subsets of all hypoglycemic AEs and all severe hypoglycemic AEs.

For tables classifying AEs by severity, if a subject has multiple occurrences of an AE with the same MedDRA term, the most severe event will be chosen.

A summary and by-subject listing will be provided for any subject who died, experienced SAEs, or experienced AEs resulting in discontinuation of study drug or the study.

Non‑treatment-emergent AEs will not be summarized.

### 6.5.2 Clinical Laboratory Evaluation

Clinical laboratory results will be tabulated. Values outside of the normal ranges will be identified and reported.

### 6.5.3 Vital Signs

For each assessment of vital signs, changes in vital signs from baseline (Day 0 pre-dose) will be summarized. Vital signs (including include pulse, blood pressure [systolic/diastolic], respiratory rate, temperature [oral or tympanic]), p0_2_, body weight, height, and BMI will be summarized by treatment group and assessment time.

## 6.6 Missing Data

Safety results will be summarized using all available data. Multiple attempts will be made to obtain missing data. If there is missing data affecting the primary endpoint, a sensitivity analysis using last observation carried forward (LOCF) will be conducted.

## 6.7 Interim Analyses

There is no formal interim analysis for this study.

## 6.8 Publication Policy

The study will be sought published in an international peer-reviewed journal with the TNIL6 Study Group as author. Both positive and negative results will be sought published.

# 7.0 quality control and quality assurance

## 7.1 Ethical Considerations – Overall Risk and Benefit

This study will be conducted in accordance with the ethical principles stated in the most recent version of the Declaration of Helsinki II and the applicable guidelines on good clinical practice, whichever affords the greater protection to the individual.

As mentioned IL-1 antagonism with anakinra has been widely used as part of clinical practice in rheumatoid arthritis in more than 100.000 patients (8,9). Adverse event rates did not statistically exceed control levels in placebo-controlled trials.

There are no further risks to this study, besides the already mentioned drug related ones.

For the subjects participating in this study there will be, apart from the financial compensation, no further benefit.

## 7.2 Informed Consent

The informed consent document will be signed by the subject or the subject’s legally authorized representative before the subject participates in the study. Signed consent forms will be maintained as part of the study file and will be available for verification at any time. A copy of the informed consent document will be provided to the subject or the subject’s legally authorized representative

## 7.3 Study Monitoring Requirements

The CTU will be monitoring this study. Authorized representatives of the national or local health authorities will be permitted to inspect or audit the facilities and records relevant to this study.

## 7.4 Disclosure of Data

Subject medical information obtained for this study is confidential, and disclosure to third parties other than those noted below is prohibited. Subject data will be identified by study, subject ID number and initials.

Upon the subject’s permission, medical information may be given to his or her personal physician or other appropriate medical personnel responsible for his or her welfare.

Data generated by this study must be available for inspection upon request by representatives of the appropriate national and local health authorities, and the EC for each study site, if appropriate.

## 7.5 Retention of Records

Records and documents pertaining to the conduct of this study, including CRFs, consent forms, laboratory test results, clinical notes, and medication inventory records, will be retained for 10 years

# 8.0 REFERENCES

1. Spranger J, Kroke A, Mohlig M, Hoffmann K, Bergmann MM, Ristow M, Boeing H, Pfeiffer AF (2003): Inflammatory cytokines and the risk to develop type 2 diabetes: results of the prospective population-based European Prospective Investigation into Cancer and Nutrition (EPIC)-Potsdam Study. *Diabetes* 52:812-817

2. Hu FB, Meigs JB, Li TY, Rifai N, Manson JE (2004): Inflammatory markers and risk of developing type 2 diabetes in women. *Diabetes* 53:693-700

3. Kristiansen OP, Mandrup-Poulsen T (2005): Interleukin-6 and diabetes: the good, the bad, or the indifferent? *Diabetes* 54 Suppl 2:S114-124

4. Larsen CM, Faulenbach M, Vaag A, Volund A, Ehses JA, Seifert B, Mandrup-Poulsen T, Donath MY (2007): Interleukin-1-receptor antagonist in type 2 diabetes mellitus. *N Engl J Med* 356:1517-1526

5. Pedersen BK, Febbraio MA (2008): Muscle as an endocrine organ: focus on muscle-derived interleukin-6. *Physiol Rev* 88:1379-1406

6. Al-shair K, Kolsum U, Dockry R, Morris J, Singh D, Vestbo J (2011): Biomarkers of systemic inflammation and depression and fatigue in moderate clinically stable COPD. *Respir Res* 12:3

7. Main LC, Dawson B, Heel K, Grove JR, Landers GJ, Goodman C (2010): Relationship Between Inflammatory Cytokines and Self-Report Measures of Training Overload. *Res Sports Med* 18:127-39

8. Lee DL, Leite RL, Fleming C, Pollock JS; Webb C, and Brands MW (2004): Hypertensive Response to Acute Stress Is Attenuated in Interleukin-6 Knockout Mice. *Hypertension* 44:259-263

9. Brands MW, Banes-Berceli AKL, Inscho EW, Al-Azawi H, Allen AA, and Labazi H (2010): Interleukin 6 Knockout Prevents Angiotensin II Hypertension: Role of Renal Vasoconstriction and Janus Kinase 2/Signal Transducer and Activator of Transcription 3 Avtivation. *Hypertension* 56:879-884

10. Fleischmann RM, Tesser J, Schiff MH, Schechtman J, Burmester GR, Bennett R, Modafferi D, Zhou L, Bell D, and Appleton B (2006): Safety of extended treatment with anakinra in patients with rheumatoid arthritis. *Ann Rheum Dis* 65:1006-12.

11. Schiff MH, Divittorio G, Fleischmann RM, Schectman J, Hartman S, Liu T, Solinger AM (2004): The Safety of Anakinra in High-Risk Patients with Active Rheumatoid Arthritis – Six-Month Observations of Patients with Comorbid Conditions. *Arthritis & Rheumatism* 50:1752-60

12 Frey I, Berg A, Grathwohl D et al., (1999): Freiburger Fragebogen zur körperlichen Aktivität – Entwicklung, Prüfung, Anwendung

13. Mathiowetz V, Kashman N, Volland G, Weber K, Dowe M, Rogers S (1985): Grip and pinch strength: normative data for adults. *Arch Phys Med Rehabil* 66:69-74

14. L.D. Hubbard, R.J. Brothers and W.N. King *et al.*, Methods for evaluation of retinal microvascular abnormalities associated with hypertension/sclerosis in the Atherosclerosis Risk in Communities Study, *Ophthalmology* **106** (12 (December)) (1999), pp. 2269–2280.

15. Shirai K, Utino J, Otsuka K, Takata M. A novel blood pressure-independent arterial wall stiffness parameter; cardio-ankle vascular index (CAVI). J Atheroscler Thromb 2006;13(2):101-107.

16. Okura T, Watanabe S, Kurata M et al. Relationship between cardio-ankle vascular index (CAVI) and carotid atherosclerosis in patients with essential hypertension. Hypertens Res 2007;30(4):335-340.

Appendix 1
Standard Operating Procedure for the Treadmill Ergometer Test

**(**Assessing individual HR-VO_2_ relationships and VO_2_max**)**

The VO_2_max test is performed on the screening visit (day -28 to -7).

The purpose of the VO_2_max test is to determine individual heart rate (HR)-VO_2_ relationships, VO_2_max and HRmax during treadmill running.

The results from this test will be used to determine the HR corresponding to 75% of VO_2_max at which the subject will run during the 1-hour running test at visits 1, 2 and 3.

The VO_2_max test will be performed on a treadmill ergometer using an online system allowing direct measurements of O_2_ uptake.

Prior to starting the test, the subject is informed about the procedure, and before starting to warm up, a heart rate monitor and mouthpiece are placed around the chest and in the mouth respectively.

The actual test contains two parts: 1) 6 min work-out intervals at various running speed (HR-VO_2_ relationship), and 2) all out incremental VO_2_max test).

The subjects start running for 6 min at 10 km/h, followed by a 2 min break, continues for 6 min at 12 km/h, 2 min break, 6 min at 14km/h, 2 min break and 6 min at 16 km/h. After the last 6 min running period, the individual has a 10 min break and then the all out incremental test starts. The subject starts running at 16km/h for 2 min, followed by an increase by 1km/h every min until exhaustion.

Appendix 2a Research Chart

|  | Pre-screening phone call | **Screen**  (Day -28 to -7) | **Visit 1**  (Day 0) | +24h | +48h | +72h | **Visit 2**  [Day 6 (+3 Days)] | +24h | +48h | +72h | **Visit 3**  [Day 12 (+6 Days)] |
| --- | --- | --- | --- | --- | --- | --- | --- | --- | --- | --- | --- |
|  |  |  |  |  |  |  |  |  |  |  |  |
| Inclusion/exclusion criteria | x | x |  |  |  |  |  |  |  |  |  |
| Informed consent |  | x |  |  |  |  |  |  |  |  |  |
| Demographics |  | x |  |  |  |  |  |  |  |  |  |
| Medical history |  | x | x |  |  |  |  |  |  |  |  |
| Concomitant medication |  | x | x |  |  |  | x |  |  |  | x |
| Physical examination[[1]](#footnote-2) |  | x |  |  |  |  |  |  |  |  | x |
| ECG |  | x |  |  |  |  |  |  |  |  |  |
| CBC |  | x |  |  |  |  |  |  |  |  | x |
| Serum chemistry[[2]](#footnote-3) |  | x |  |  |  |  |  |  |  |  | x |
| Vital signs |  | x | x |  |  |  | x |  |  |  | x |
| Measurements of R thigh and R arm |  | x |  |  |  |  |  |  |  |  |  |
| Treadmill ergometer |  | x |  |  |  |  |  |  |  |  |  |
| Adverse events |  |  | x | x | x | x | x | x | x | x | x |
| Activity quetsionnaire |  | x |  |  |  |  |  |  |  |  |  |
| Muscle soreness |  |  |  | x | x | x |  | x | x | x |  |
| ACTIF pre |  |  | x |  |  |  | x |  |  |  |  |
| ACTIF post |  |  | x |  |  |  | x |  |  |  |  |
| SDMT |  |  | x |  |  |  | x |  |  |  |  |
| BDI-FS |  |  | x |  |  |  | x |  |  |  |  |
| Hand-grip-Strength-Test |  |  | x |  |  |  | x |  |  |  |  |
| VAS Fatigue after exercise |  |  | x |  |  |  | x |  |  |  |  |
| Cavi and SVA before injection |  |  | x |  |  |  | x |  |  |  |  |
| Cavi and SVA directly after exercise |  |  | x |  |  |  | x |  |  |  |  |
| Cavi and SVA 1 hour after exercise |  |  | x |  |  |  | x |  |  |  |  |
| **Exercise bout** |  |  | x |  |  |  | x |  |  |  |  |
|  |  |  |  |  |  |  |  |  |  |  |  |
| **Trial medication** |  |  |  |  |  |  |  |  |  |  |  |
| Anakinra |  |  | x |  |  |  | x |  |  |  |  |
| placebo |  |  | x |  |  |  | x |  |  |  |  |

| **Blood analysis** |  |  |  |  |  |  |  |  |  |  |  |
| --- | --- | --- | --- | --- | --- | --- | --- | --- | --- | --- | --- |
| IL-6 |  |  | x |  |  |  | x |  |  |  |  |
| GLP-1 (active) |  |  | x |  |  |  | x |  |  |  |  |
| Glucagon |  |  | x |  |  |  | x |  |  |  |  |
| Insulin |  |  | x |  |  |  | x |  |  |  |  |
| Glucose |  |  | x |  |  |  | x |  |  |  |  |
| IL-1Ra |  |  | x |  |  |  | x |  |  |  |  |
| GIP |  |  | x |  |  |  | x |  |  |  |  |
| CRP |  |  | x |  |  |  | x |  |  |  |  |
| TNF-a |  |  | x |  |  |  | x |  |  |  |  |
| Cortisol |  |  | x |  |  |  | x |  |  |  |  |
| GH |  |  | x |  |  |  | x |  |  |  |  |
| Retro Samples |  |  | x |  |  |  | x |  |  |  | x |
| CK |  | x | x |  |  |  | x |  |  |  | x |
| **Urinanalysis** |  | x |  |  |  |  |  |  |  |  |  |

Appendix 2b – Research Chart Footnotes

^2^Physical examinations includes:

Status of Heart and Lungs

Registration of right thigh (15cm above patella) and right upper arm (10cm above epicondylus lateralis) diameter in order to standardize muscle mass

^2^ Serum chemistry includes:

Sodium

Potassium

creatinine

CRP

liver transaminases (ALT,AST)

Glucose

AP

Appendix 3

Standard Operating Procedure for the 1-hour Treadmill Ergometer Exercise

Subjects are fasted 6 to 10 hours prior to the visit.

An intravenous catheter for blood drawings will be placed in the forearm/elbow, 5 minutes prior to first time point for blood drawing. 60 minutes before exercise, the first blood sample will be drawn. Right after the first blood sample has been taken, subjects will receive a single subcutaneous injection of **100mg anakinra or placebo** (crossover from Visit 1). Between blood draws, the IV catheters will be kept patent by a slow infusion of saline. The 1-hour treadmill exercise begins 60min after drug/placebo injection.

Prior to starting the 1-hour exercise the subject is informed about the procedure. A heart rate monitor is placed around the chest and the subject begins to run starting with a 5 min warm up period at 2 to 4 km/h under target speed at an incline of 0.5%. After 5 min, and in continuation of warm up, treadmill speed is increased so the subject reaches 75% to 85% of VO_2max_ based on heart rate measurements, and this relative workload is maintained for the remaining time (approx. 55 min, total running time 60 min).

For blood sampling, we will lower the speed of the treadmill to walking speed in order to be able to draw the blood without any complications.

After 60 min running, the subject will stay on the treadmill for a “cool down” at walking speed for 5 min. For the remaining time until finishing with the blood drawing, subjects will be seated on a chair.

After the last time point for blood drawing (60 minutes after exercise) the intravenous catheter will be removed. Since subjects have been fasting prior to the exercise bout, we will provide them with a sandwich and a cold drink after finishing with the last blood drawing. If, 15minutes after the last blood drawing, subjects show no signs of malaise they will be given information concerning their next exercise bout and are free to go.

Appendix 4
Adverse event definition and reporting guidelines

**Adverse Event (AE):** Any untoward medical occurrence in a trial subject administered a pharmaceutical product and which does not necessarily have a causal relationship with this product. An AE can therefore be any unfavorable and unintended sign (including an abnormal laboratory finding), symptom, or disease temporally associated with the use of a pharmaceutical study product, whether or not related to the pharmaceutical study product.

AEs observed by the sponsor/investigator and/or reported by the subject must be reported in the source data and CRF during the entire study period, i.e. the period of time from the first (= signature of informed consent) to the last protocol-specific procedure regardless of the medicinal study product relation assessment.

The “General Definitions of Severity Grades” are:

- *Mild* (grade 1): The AE is noticeable to the subject but does not interfere with subject’s every day’s life activities; it may or may not require additional concomitant therapy.
- *Moderate* (grade 2): The AE interferes with the subject’s daily activities; it usually requires additional therapy.
- *Severe* (grade 3): The AE is intolerable and requires additional therapy.
- *Life threatening* (grade 4): The individual is at immediate risk of death at the time of the AE; it does not refer to an event which hypothetically might have caused death if it was more severe. Life-threatening events result in Serious Adverse Events (SAE).

**Serious Adverse Event (SAE):** Any untoward medical occurrence medical occurrence that at any dose

- results in death,
- is life-threatening,
- requires subject hospitalization or prolongation of current hospitalization,
- results in persistent or significant disability/incapacity, or congenital anomaly/birth defect.

Furthermore any important medical event and any event which, though not included in the above, may jeopardize the subject or may require intervention to prevent one of the outcomes listed above.

Recording of (Serious) Adverse Events: Clinical study subjects will be routinely questioned about AEs at study visits. The well-being of the subjects will be ascertained by neutral questioning (e.g. "How are you?"). The sponsor/investigator is responsible for reporting all AEs occurring during the course of the study.

All observed or volunteered adverse drug events and abnormal test findings will be recorded in the source data and CRF.

AEs or abnormal test findings felt to be associated with the study treatment(s) will be followed until the event (or its sequelae) or the abnormal test finding resolves or stabilizes at a level acceptable to the sponsor/investigator.

An abnormal test finding will be classified as an AE if one or more of the following criteria are met:

- The test finding is accompanied by clinical symptoms.
- The test finding necessitates additional diagnostic evaluation(s) or medical/surgical intervention; including significant additional concomitant drug treatment or other therapy (Simply repeating a test finding, in the absence of any of the other listed criteria, does not constitute an AE.).
- The test finding leads to a change in study dosing or discontinuation of subject participation in the clinical study.

All (S)AEs will be fully documented in the source data and on the appropriate pages of the CRF. For each (S)AE, the sponsor/investigator will provide onset, duration, intensity, treatment required, outcome and action taken with regard to the investigational products/treatment.

The sponsor/investigator will determine the relationship of the investigational products/treatment to all AEs as defined on the *Adverse Event Reporting Form*.

Assessment of (Serious) Adverse Events: The sponsor/investigator will promptly review documented AEs and abnormal test findings to determine if

- the abnormal test finding should be classified as an AE,
- if there is a reasonable possibility that the AE was caused by the administered pharmaceutical product, and
- if the AE meets the criteria for an SAE.

Using the following criteria the sponsor/investigator also needs to assess whether there is a reasonable possibility that the administered pharmaceutical product caused or contributed to the AE:

- Yes, *possibly, probably* or *definitely related*
- There is a clinically plausible time sequence between onset of the AE and administration of pharmaceutical product. AND/OR
- There is a biologically plausible mechanism for pharmaceutical product causing or contributing to the AE.
- No, *unlikely related* or *unrelated*
- Another cause of the AE is most plausible. AND/OR
- A clinically plausible temporal sequence is inconsistent with the onset of the AE and administration of pharmaceutical product. AND/OR
- A causal relationship is considered biologically implausible.

Reporting of Serious Adverse Events: The sponsor/investigator is responsible for SAE reporting to Swissmedic and to the IEC, respectively, according to the following details:

- The Sponsor/Investigator is responsible for:
- Compliance with the regulatory requirements of Swissmedic regarding prompt reporting of unexpected SAEs for which a causal relationship with the study drug or device cannot be ruled out.
- Reporting to Swissmedic of fatal and life-threatening SAEs if evaluated as Suspected Unexpected Serious Adverse Reaction (SUSAR):
- *without delay* and *no later than 7 calendar days* following awareness that event meets criteria for a SUSAR;
- follow-up information regarding the SUSAR within *further 8 calendar days*
- Reporting to Swissmedic of non-fatal and not life-threatening SAEs if evaluated as “suspected”, “unexpected” and “drug related” (SUSARs)
- *promptly* and no later than *15 calendar days* following awareness that event meets criteria for a SUSAR.
- Sending yearly safety reports, starting one year after the date of notification to Swissmedic. These reports should contain:
- A listing of all SUSARs that have occurred in Switzerland and at international level (if applicable).
- Reporting to IEC any SAE which resulted in death:
- immediately, i.e. *within 24 hours.*

An unexpected SAE refers to any AE, the nature or severity of which is not consistent with the applicable product information, namely *please specify for unapproved investigational pharmaceutical products the current Investigator’s Brochure and the* *approved label for registered drugs.*

Reporting of Pregnancy: Subjects, whose women become pregnant during the trial have to stop further treatment immediately.

Pregnancy should be confirmed by a reliable laboratory test. Subjects whose women are pregnant must be immediately withdrawn from the clinical study. All pregnancies occurring during the treatment phase of the study and within 30 days after discontinuation of study drug have to be reported to the Sponsor within one working day of the investigational sites knowledge of the pregnancy on the *Pregnancy Report Form*. However, if the termination visit takes place more than 30 days after the subject has discontinued study drug, the reporting time has to be extended until the termination visit. The Sponsor will contact the attendant physician by phone during pregnancy and after the estimated date of delivery to enquire about course and outcome of the pregnancy. Course of the pregnancy and health status of the new born child have to be documented and sent to the pharmaceutical product manufacturer(s).

Reporting of Hypoglycemia: Is classified according to the American Diabetes Association (ADA) workgroup on hypoglycaemia as follows:

- *Severe hypoglycaemia*: Event requiring assistance of another person to actively administer carbohydrate, glucagon, or other resuscitative actions. These episodes may be associated with sufficient neuroglycopenia to induce seizure or coma. Plasma glucose measurements may not be available during such an event, but neurological recovery attributable to the restoration of plasma glucose to normal is considered sufficient evidence that the event was induced by a low plasma glucose concentration.
- *Documented symptomatic hypoglycaemia*: Defined as an event during which typical symptoms of hypoglycaemia are accompanied by a measured plasma glucose concentration less than or equal to 2.5mmol/L.
- *Documented asymptomatic hypoglycaemia*: An event not accompanied by typical symptoms of hypoglycaemia, but with a measured plasma glucose concentration less than or equal to 2.5mmol/L.
- *Probable symptomatic hypoglycaemia*: Defined as an event during which typical symptoms of hypoglycaemia are not accompanied by a plasma glucose determination, but was presumably caused by a plasma glucose concentration less than or equal to 2.5mmol/L. Diabetic subjects will be instructed to make every effort to document suspected hypoglycaemia.
- *Relative hypoglycaemia*: Defined as an event during which the person with diabetes reports any of the typical symptoms of hypoglycaemia, and interprets the symptoms as indicative of hypoglycaemia, but with a measured plasma glucose concentration greater than 2.5mmol/L.

All above mentioned events concerning diabetic subjects should be reported and documented on the hypoglycaemia report in the CRF.

Reporting of Serious Adverse Events: The sponsor/investigator is responsible for SAE reporting to Swissmedic and to the IEC, respectively, according to the following details:

- The sponsor is responsible for:

Follow-up of (Serious) Adverse Events

Subjects terminating the study (either regularly or prematurely) with:

- reported ongoing SAE, or
- any ongoing AEs of laboratory values or of vital signs being beyond the alert limit

will return for a follow-up investigation. This visit will take place up to 30 days after terminating the treatment period. Follow-up information on the outcome will be recorded in the source date and on the respective AE page in the CRF.

For any AEs the outcome "unknown" is not acceptable, except if attempts to collect the information have been made and documented. In case of subjects lost to follow-up, efforts should be made and documented to contact the subject to encourage him/her to continue study participation as scheduled. In case of minor AEs a telephone call to the subject may be acceptable.

All new SAE or pregnancies that the investigators will be notified of within 30 days after discontinuation of study drug will be reported in the source data and in the CRF if required. However, if the termination visit takes place more than 30 days after the subject has discontinued study drug, the reporting time has to be extended until the termination visit.

Follow-up investigations may also be necessary according to the sponsor’s/investigator’s medical judgment even if the subject has no AE at the end of the study. However, information related to these investigations does not have to be documented in the CRF but must be noted in the source documentation.

**Appendix 5
Activity Induced Fatigue (ACTIF) Scale before and after exercise**

**Appendix 6
Beck-depression-inventory Fast Screen (BDI-FS)**

**Appendix 7
Symbol Digit Modalities Test (SDMT)**

**Appendix 8
VAS – Fatigue after exercise**

**Appendix 9
Hand-Grip-Strength-Test**

The Hand-Grip-Strength-Test is assessing the maximal isometric muscle strength of the hand and the arm.

This test is performed using a calibrated dynamometer. The subject is told to squeeze the dynamometer with one hand as hard as possible. Maximal strength is read off from the dynamometer by the investigator. The subject is squeezing the dynamometer 3 times in a row with each hand and the best result from each hand is counted (13).

**Appendix 10**

**Retinal microcirculation and the arteriolar to venular ratio (AVR)**

Analysis will be performed using the Static Retinal Vessel Analyzer (SVA-T, Imedos Systems UG, Jena, Germany). The system allows non-invasive online measurement of the diameter of retinal vessels without mydriasis. It consists of a fundus camera and an advanced image processing unit (14).

For analysis, 3 valid images are taken from the retina of the left and right eye, with an angle of 30° and the optic disc in the center. Retinal arterioles and venules, coursing through an area of 0.5–1 disc diameter from the margin of the optic disc, will be identified using special analyzing software identifying retinal vessels in ring-zones (Vesselmap 2, Visualis, Imedos Systems UG). A detailed and standardized evaluation of retinal vessel diameters will be performed on higher-magnification images. All retinal arterioles and venules will be differentiated by the examiner in the outer ring zone and measured by the automated software. Vessel diameters below ≤45 μm will be generally discarded. Diameters will be calculated to central retinal arteriolar and venular equivalents (CRAE, CRVE), using the Parr-Hubbard formula described elsewhere (14). The CRAE and CRVE are used to calculate the arteriolar-to-venular-ratio (AVR), taking the mean of the right and left eye results. Vessel diameters are presented in measuring units (mu). In the model of Gullstrand‘s normal eye, 1 mu relates to 1 μm. The assessment of the retinal vessels will be performed by a single experienced examiner.

**Appendix 11**

**Cardio-ankle vascular index (CAVI) and Pulse wave velocity (PWV)**

The measurement of large arterial stiffness as cardio-ankle vascular index (CAVI) is a reliable mean of measurement of arterial stiffness with good reproducibility (15,16). CAVI is an index of arterial stiffness and is calculated as a × [(2ρ/ΔP)×(ln SBP/DBP)×(PWV)2]+b×(a,b constant;ρ,blood density; ΔP, difference in systolic and diastolic pressure). CAVI is calculated by an oscillometric sphygmograph (VaSera VS-1500; Fukuda Denshi, Tokyo, Japan). CAVI reflects the stiffness of the aorta, femoral artery and tibial artery as a whole. It is theoretically not affected by blood pressure. All measurements are made automatically by the VS-1500 device. For measurement, cuffs will be applied to both upper arms and ankles, with the subject lying supine. After resting for 10 min, the examination will be performed. To detect the brachial and ankle pulse waves with the cuffs, a low cuff pressure of 30–50mmHg will be used to ensure minimal effect of cuff pressure on hemodynamics. Pulse wave velocity from the heart to the ankle will be obtained by measuring the length from the aortic valve to the ankle.

**Appendix 12**

**Activity questionnaire (Aktivitätsfragebogen)**

1. [↑](#footnote-ref-2)
2. [↑](#footnote-ref-3)
